# Targeting eIF4A-dependent translation in genetically complex sarcoma

**DOI:** 10.1172/jci.insight.192936

**Published:** 2026-04-07

**Authors:** Young-Mi Kim, Prathibha Mohan, Urmila Sehrawat, Evan Seffar, Rafaela Muniz De Queiroz, Kalyani Chadalavada, Nikita Persaud, Tomoyo Okada, Anirudh Kulkarni, Jianan Lin, Nathalie Lailler, Shaleigh Smith, Bhumika Jadeja, Nicholas D. Socci, Zhengqing Ouyang, Hans-Guido Wendel, Samuel Singer

**Affiliations:** 1 Sarcoma Biology Laboratory,; 2 Department of Surgery,; 3 Sarcoma Center, and; 4 Cancer Biology & Genetics Program, Sloan Kettering Institute, Memorial Sloan Kettering Cancer Center (MSK), New York, New York, USA.; 5 The Jackson Laboratory for Genomic Medicine, Farmington, Connecticut, USA.; 6 Marie-Josée and Henry R. Kravis Center for Molecular Oncology and; 7 Bioinformatics Core, MSK, New York, New York, USA.; 8 Department of Biostatistics and Epidemiology, School of Public Health and Health Sciences, University of Massachusetts, Amherst, Massachusetts, USA.

**Keywords:** Cell biology, Oncology, Oncogenes, Translation

## Abstract

Dedifferentiated liposarcoma (DDLS), myxofibrosarcoma (MFS), and undifferentiated pleomorphic sarcoma (UPS) are the most common types of genetically complex sarcoma. There is an urgent need to develop effective targeted therapy for these deadly sarcoma types. Despite their genetic complexity, these sarcomas share genomic alterations causing PI3K/Akt/mTOR and MAPK pathway activation, and both pathways control translation mediated by the RNA helicase eIF4A. We therefore investigated eIF4A inhibition as a therapeutic strategy. The eIF4A inhibitor CR-1-31B effectively suppressed tumor growth and induced apoptosis in DDLS, MFS, and UPS patient–derived cell lines and mouse xenografts. Transcriptome-scale ribosome footprinting identified eIF4A-dependent mRNAs such as the Hippo pathway transcriptional coactivators *YAP1* (YAP) and *WWTR1* (TAZ). Combined knockdown of YAP and TAZ induced apoptosis in DDLS, MFS, and UPS cell lines, and their ectopic expression partially rescued cells from apoptosis induced by CR-1-31B. Genomic analysis of patient tumors revealed that *YAP* and *WWTR1* were frequently amplified or gained in DDLS, MFS, and UPS and were associated with worse clinical outcomes. Together, our findings identify a strategy for targeting the Hippo pathway in incurable forms of sarcoma based on inhibition of eIF4A-dependent translation of the key oncogenic transcription factors YAP and TAZ.

## Abstract 2

This study demonstrates that inhibiting eukaryotic initiation factor 4A, required for translation of oncogenic transcription factors YAP and TAZ, suppresses growth of incurable sarcomas.

## Introduction

Myxofibrosarcoma (MFS), undifferentiated pleomorphic sarcoma (UPS), well-differentiated liposarcoma (WDLS), and dedifferentiated liposarcoma (DDLS) are the most common and deadly types of genetically complex sarcoma. WDLS and DDLS are characterized by 12q amplification, encompassing the oncogenes *CDK4*, *MDM2*, and *HMGA2* in > 95% of cases. WDLS is a locally aggressive, rarely metastasizing, malignant mesenchymal neoplasm composed of proliferating mature adipocytes with considerable variation in cell size and nuclear atypia. DDLS is a high-grade, clinically aggressive tumor that is almost always associated with an adjacent region of WDLS and contains a region of nonlipogenic sarcoma in at least 20% of the tumor. MFS and UPS are associated with copy number deletions or loss-of-function mutations in *RB1* and *TP53* in 70% of cases (1–4). The development of new targeted therapies is vital to improve patients’ outcomes. However, the complexity of genetic alterations in these sarcomas has made it difficult to find and target the true drivers of oncogenesis. More than 60% of patients with retroperitoneal WDLS and DDLS eventually die of disease despite complete surgical resection (5). Approximately 50% of patients with UPS and MFS develop lung metastasis and die of disease despite multimodality therapy (6). Moreover, chemotherapy for advanced disease is associated with substantial toxicity and low response rates. Thus, there is an urgent need to identify critical vulnerabilities and develop effective targeted treatments for patients with these types of genetically complex sarcomas.

Previous studies have shown that MFS, UPS, and DDLS share genomic alterations that result in PI3K/Akt/mTOR and MAPK pathway activation (7–13), the 2 predominant signaling cascades that regulate cell growth, proliferation, and survival. Signaling via mTOR and MAPK converge to regulate protein synthesis through the eIF4F translation initiation complex, composed of the DEAD-box RNA helicases eIF4A, the cap binding protein eIF4E, and the regulatory scaffold protein eIF4G (14). The RNA helicase component of this complex is required to unwind secondary structure in the 5′-UTR and initiate translation of mRNAs, specifically those RNAs whose 5′-UTRs contain complex structures such as RNA G-quadruplexes (15–17). Importantly, these mRNAs include key oncogenes such as *MYC*, *BCL2*, *CDK6*, *KRAS*, and *YAP1* (15–17), which are critical for tumorigenesis.

The eIF4A gene encodes 2 isoforms, eIF4A1 and eIF4A2, which share approximately 90% sequence similarity and can both integrate into the eIF4F complex. While these isoforms are thought to perform similar roles in translation initiation, they also exhibit distinct functions in vivo. Specifically, eIF4A1 is essential for embryonic development in mice (18) and in various cancer cell lines (19), whereas eIF4A2 is not. eIF4A inhibitors, which target both isoforms, suppress translation of key oncogenes and have demonstrated antitumor activity in a variety of cancers (15, 17, 19–22).

In DDLS, UPS, and MFS, the Hippo pathway is frequently deregulated through increased expression of the transcriptional coactivators YAP and TAZ (9, 23–25). The Hippo pathway controls the YAP-TAZ-TEAD transcriptional complex that promotes the expression of genes important for cancer cell proliferation, survival, and progression (26–28). Moreover, Hippo signaling regulates adipocyte proliferation and differentiation (29, 30), and TAZ represses PPARγ-dependent transcription, thereby opposing adipocyte differentiation and modulating mesenchymal stem cell differentiation (31). These functions make the oncogenic YAP-TAZ-TEAD complex an attractive cancer target, but no therapeutics that can block its activity have been developed.

A synthetic eIF4A inhibitor, CR-1-31B, has shown potent activity against many cancer cell lines and promising efficacy in several mouse models (15, 17, 20, 21, 32, 33). Therefore, we investigated eIF4A inhibition as a therapeutic strategy against these sarcomas. In this study, we show that MFS, UPS, and DDLS rely on oncogenic translation enabled by the RNA helicase eIF4A for growth and survival and use ribosome profiling to identify *YAP1* and *WWTR1* (TAZ) as critical translational targets of the eIF4A inhibitor, CR-1-31B.

## Results

### Inhibition of eIF4A reduces proliferation and increases apoptosis in DDLS cells.

**Figure 1:**
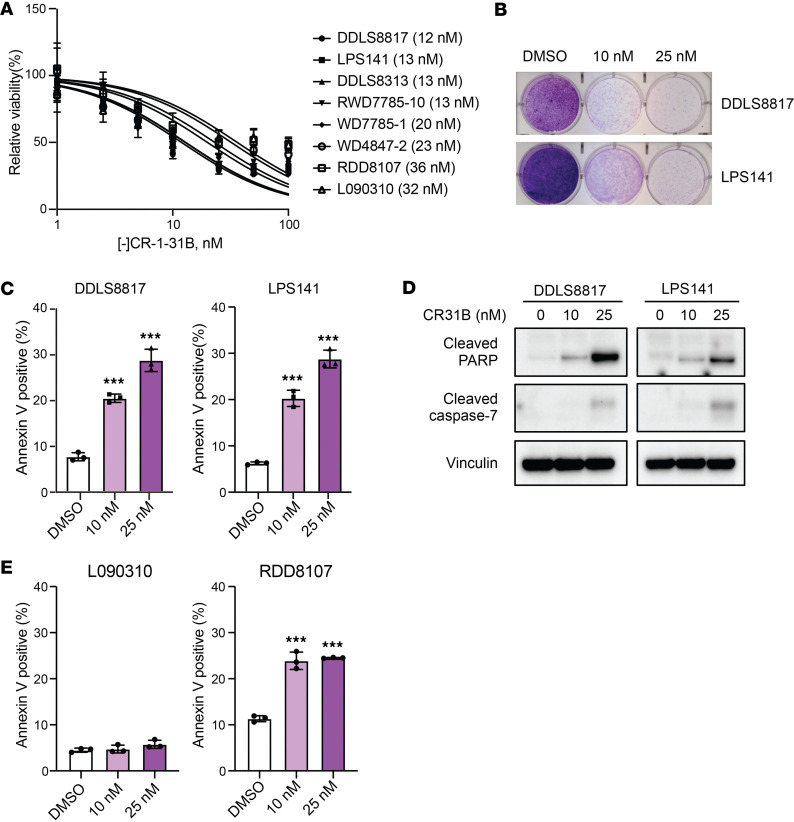
[-]CR-1-31B is active against a panel of WD/DDLS cell lines. (**A**) Proliferation of WD/DDLS cells and normal ASCs (L090310) incubated with the indicated concentrations of [-]CR-1-31B for 72 hours as assessed by CyQUANT assay. (**B**) Colony formation of DDLS cells treated with [-]CR-1-31B or vehicle (DMSO) for 48 hours followed by culture in media without drug for 10 days. Colonies stained with crystal violet. (**C** and **D**) Apoptosis of DDLS cells treated with [-]CR-1-31B for 72 hours as measured by annexin V and 7-AAD costaining (**C**), and expression of the apoptosis markers cleaved PARP and cleaved caspase-7 in DDLS cells treated with [-]CR-1-31B for 72 hours (**D**). Vinculin was used as a loading control. (**E**) Apoptosis as measured by annexin V and 7-AAD costaining in L090310 and RDD8017 treated for 72 hours with [-]CR-1-31B. ***P < 0.001 by 1-way ANOVA.

We examined the activity of the active [-] enantiomer of the eIF4A inhibitor CR-1-31B (17) against a panel of WD/DDLS cell lines and observed low nanomolar IC_50_ values at 72 hours by CyQUANT assay (Figure 1A). Accordingly, 10 nM [-]CR-1-31B substantially inhibited colony formation (Figure 1B). As the CyQUANT assay does not distinguish cytotoxicity from cytostasis, we evaluated whether [-]CR-1-31B induces cell death by staining with annexin V and 7-AAD, which confirmed apoptosis in DDLS8817 and LPS141 cells (Figure 1C). Consistent with these results, we detected increased PARP cleavage and caspase-7 activation in [-]CR-1-31B–treated cells by Western blot (Figure 1D).

[-]CR-1-31B also inhibited the growth of normal adipose-derived stem cells L090310 with an IC_50_ of 32 nM, which is comparable with its effects on RDD8107 cancer cells (IC_50_ = 36 nM) (Figure 1A). However, treatment with 25 nM [-]CR-1-31B induced apoptosis in RDD8107 but not in L090310, indicating that [-]CR-1-31B exerts a cytotoxic effect specifically in cancer cells (Figure 1E).

[-]CR-1-31B equally targets both eIF4A isoforms, eIF4A1 and eIF4A2, which share 90% amino acid similarity (34). To determine which isoform is responsible for [-]CR-1-31B’s effects, we used siRNA to knock down eIF4A1 and eIF4A2 alone or in combination, validated by Western blot (Supplemental Figure 1A; supplemental material available online with this article; https://doi.org/10.1172/jci.insight.192936DS1). eIF4A1 depletion induced increased expression of eIF4A2 (Supplemental Figure 1A), which has previously been observed (35). Knockdown of eIF4A1 had a modest effect on proliferation, whereas no effect was observed with knockdown of eIF4A2 (Supplemental Figure 1B). However, combined knockdown of eIF4A1 and eIF4A2, significantly reduced proliferation and increased apoptosis in both DDLS8817 and LPS141 cells (Supplemental Figure 1, C and D). Together, these results indicate that eIF4A2 can compensate for loss of some functions of eIF4A1 and that inhibition of both eIF4A isoforms produces the strongest antitumor effects.

### Ribosome profiling identifies eIF4A-dependent mRNAs in DDLS.

**Figure 2:**
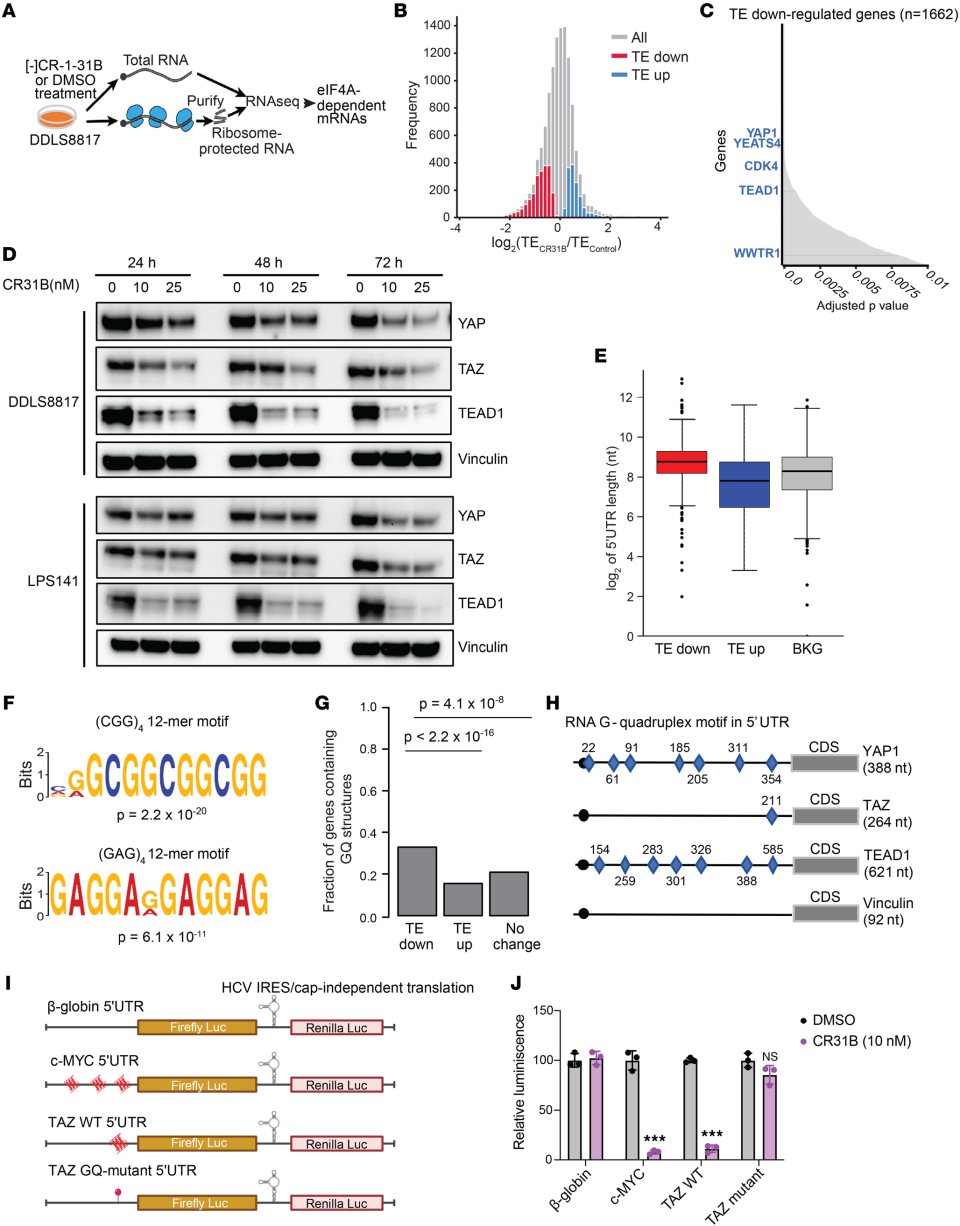
Ribosome footprinting identifies eIF4A-dependent mRNAs in DDLS cells. (**A**) Schematic of the ribosome footprinting assay. (**B**) Frequency distribution of changes in translation efficiency (TE) between control and [-]CR-1-31B–treated samples. *n* = 3 replicates. (**C**) Genes for which TE was decreased by [-]CR-1-31B ranked by significance (*q* < 0.01). (**D**) Western blots for YAP, TAZ, and TEAD1 in DDLS cells treated with [-]CR-1-31B. (**E**) Comparison of 5′-UTR lengths between TE upregulated, background, and TE downregulated genes. Statistical significance determined by Wilcoxon rank-sum test. (**F**) Two 12-mer (CGG)4 and (GAG)4 sequences significantly enriched in genes for which TE was decreased by [-]CR-1-31B. Relative letter size indicates the frequency of each amino acid. (**G**) Proportion of genes containing predicted 5′-UTR G-quadruplex (GQ) structures in genes for which TE was decreased, increased, and unaffected by [-]CR-1-31B. Statistical significance determined by Fisher’s exact test. (**H**) Positions of GQ sequences in the 5′-UTRs of *YAP*, *TA*Z, and *TEAD1* mRNAs; vinculin included as a comparison. (**I**) Schematic representation of the dual-luciferase reporter vector showing gene-specific 5′-UTR–mediated firefly luciferase expression. The 4 dual-luciferase reporters represent the 5′-UTR of β-globin (negative control), c-MYC (positive control), TAZ WT (with 1 GQ close to the AUG), and TAZ mutant with a mutated GQ, respectively. HCV IRES-driven expression of Renilla luciferase is independent of eIF4A and is used to normalize transfection efficiencies. (**J**) Effects of CR31B on Firefly luciferase activity using the reporters in **I**. Statistical significance determined by unpaired 2-tailed Student’s *t* test. ***P < 0.001.

To identify mRNAs whose translation in sarcoma cells depends on eIF4A activity, we assessed the acute effects of [-]CR-1-31B (10 nM, 2 hours) treatment on mRNA ribosome occupancy as a measure of translation efficiency (TE) in DDLS8817 cells (Figure 2A). The total number of ribosome-protected RNA fragments (ribosome footprint [RF] reads) mapped to exons was 4.5 million in control (DMSO-treated cells) and 3.1 million in [-]CR-1-31B–treated cells, corresponding to 17,930 protein coding genes. A full list of genes whose TE was differentially affected by [-]CR-1-31B in DDLS8817 cells is provided in Supplemental Table 1. With a cutoff at *q* < 0.01, we identified 1,638 mRNAs whose translation was decreased and 887 mRNAs whose translation was increased (Figure 2B and Supplemental Table 2, A and B).

Transcripts for which TE was decreased most significantly (*q* < 0.01) included *CDK4* and *YEATS4*, which are frequently amplified in DDLS, and Hippo pathway effectors including *YAP1*, *WWTR1*, and *TEAD1* (Figure 2C). Specifically, TE of YAP1 was reduced by 48% at *q* < 0.001, WWTR1 TE decreased by 28% at *q* = 0.008, and TEAD1 TE was downregulated by 33% at *q* < 0.001 (Supplemental Table 2A). Notably, mRNA levels of these genes were not significantly altered by [-]CR-1-31B, indicating that the observed TE differences were primarily due to decreased translation. Consistently, individual RF distribution graphs showed that [-]CR-1-31B treatment resulted in a loss of ribosome coverage across the 5′-UTRs and coding sequences (CDSs) of *YAP1*, *WWTR1* (encoding TAZ), and *TEAD1* (Supplemental Figure 2A). We confirmed the effects of [-]CR-1-31B on YAP, TAZ, and TEAD1 protein expression by Western blot in DDLS8817 and LPS141 cells (Figure 2D). mRNA levels of these genes were either not significantly changed or increased (Supplemental Figure 2B); increased mRNA levels may represent an autoregulatory mechanism to compensate for decreased protein expression. To rule out proteasomal degradation as a cause of the decrease in YAP, TAZ, and TEAD1 expression, we treated cells with the proteasome inhibitor MG132 in combination with [-]CR-1-31B, which did not restore expression of any of the 3 proteins in DDLS8817 or LPS141 cells (Supplemental Figure 2C).

We compared the lists of genes whose TE were increased, decreased, or unaffected by [-]CR-1-31B, and noticed that genes with decreased TE had significantly longer 5′-UTRs (TE down vs. Bkg *P* < 2.2 × 10^–16^; TE down vs. TE up *P* < × 10^–16^ vs. each of the other groups) (Figure 2E). Next, we applied the MEME motif-based sequence analysis tool (36) to investigate sequence elements that were enriched in genes whose TE was decreased by [-]CR-1-31B, which identified 2 significantly enriched 12-mer sequences (CGGCGGCGGCGG and GAGGAGGAGGAG; p = 2.2 × 10^–20^ and p = 6.1 × 10^–11^, respectively; Figure 2F). G-quadruplex (GQ) sequences were significantly enriched in the 5′-UTRs of TE down transcripts (Figure 2G). Using QGRS mapper, we identified 7 GQ sequence elements in the 5′-UTRs of *YAP1*, 1 in *TAZ*, and 7 in *TEAD1* (Figure 2H). To confirm that [-]CR-1-31B inhibits translation of genes containing GQ sequence elements in the 5′-UTR, DDLS8817 cells were transfected with TAZ-WT or TAZ-mutant reporters (Figure 2I). Treatment with 10 nM [-]CR-1-31B inhibited translation of the TAZ-WT 5′-UTR but had negligible effect on translation of the TAZ-mutant GQ 5′-UTR construct, in which the sequence was disrupted to prevent GQ formation (Figure 2J). We also attempted to test the role of GQ sequences in the YAP1 5′-UTR, but cloning into the dual-luciferase reporter system was unsuccessful due to its higher GC content and the presence of 7 GQs; synthesis of the YAP1 5′-UTR as a gene-block was also unsuccessful. Furthermore, while [-]CR-1-31B had no effect on translation of β-globin 5′-UTR, which lacks GQ sequences, it suppressed translation of the TAZ WT 5′-UTR (1 GQ) and c-MYC 5′-UTR (3 GQs) by 89% and 93%, respectively. Notably, the GQ in the TAZ 5′-UTR is positioned very close to the AUG start codon; this proximity markedly impairs translation in the absence of active eIF4A. Collectively, these results demonstrate that translation of mRNAs harboring GQ sequences in their 5′-UTRs is highly susceptible to eIF4A inhibition, which is consistent with our ribosome footprinting results.

### Ectopic expression of YAP or TAZ partially rescues cells from [-]CR-1-31B–induced apoptosis.

**Figure 3:**
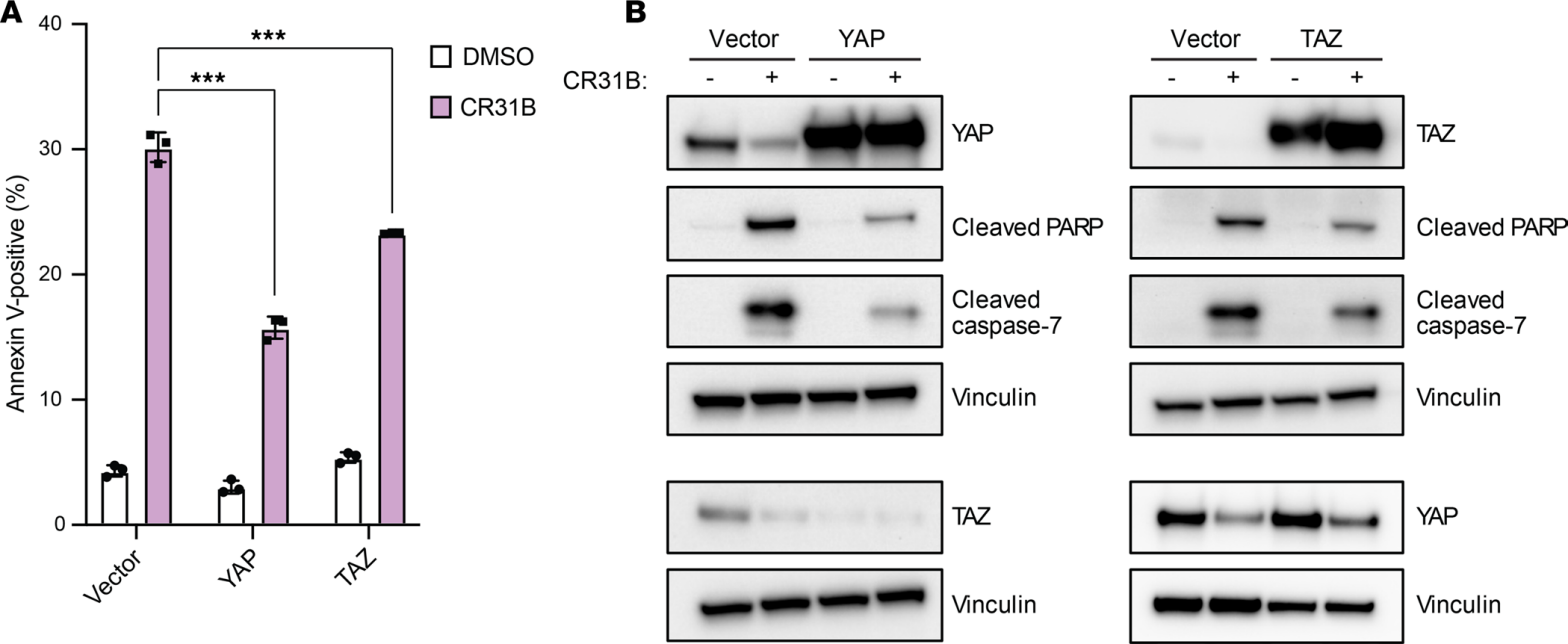
eIF4A-independent expression of YAP or TAZ partially rescues [-]CR-1-31B–induced apoptosis. DDLS8817 cells were transduced with constructs carrying YAP under the LTR promoter or TAZ under the CMV promoter. (**A** and **B**) Apoptosis, as measured by annexin V and 7-AAD costaining, and Western blots for YAP, TAZ, and the apoptosis markers cleaved PARP and caspase-7, both at 72 hours after treatment with 25 nM [-]CR-1-31B. Vinculin was used as a loading control. ***P < 0.001 by 2-way ANOVA with Tukey’s post hoc test.

As we identified YAP and TAZ as potential direct targets of eIF4A and showed that eIF4A inhibition reduces YAP and TAZ protein expression, we investigated whether YAP and TAZ are responsible for eIF4A inhibition–induced apoptosis. We performed rescue experiments in DDLS8817 cells by expressing YAP or TAZ under the LTR or CMV promoter, respectively, conferring eIF4A-independent translation. Ectopic expression of YAP or TAZ was not reduced by [-]CR-1-31B and partially rescued cells from apoptosis induced by [-]CR-1-31B (Figure 3, A and B). These results suggest that decreased expression of YAP and TAZ contributes to the effects of [-]CR-1-31B on apoptosis.

### Inhibition of eIF4A using [-]CR-1-31B reduces proliferation and induces apoptosis in MFS and UPS cells.

**Figure 4:**
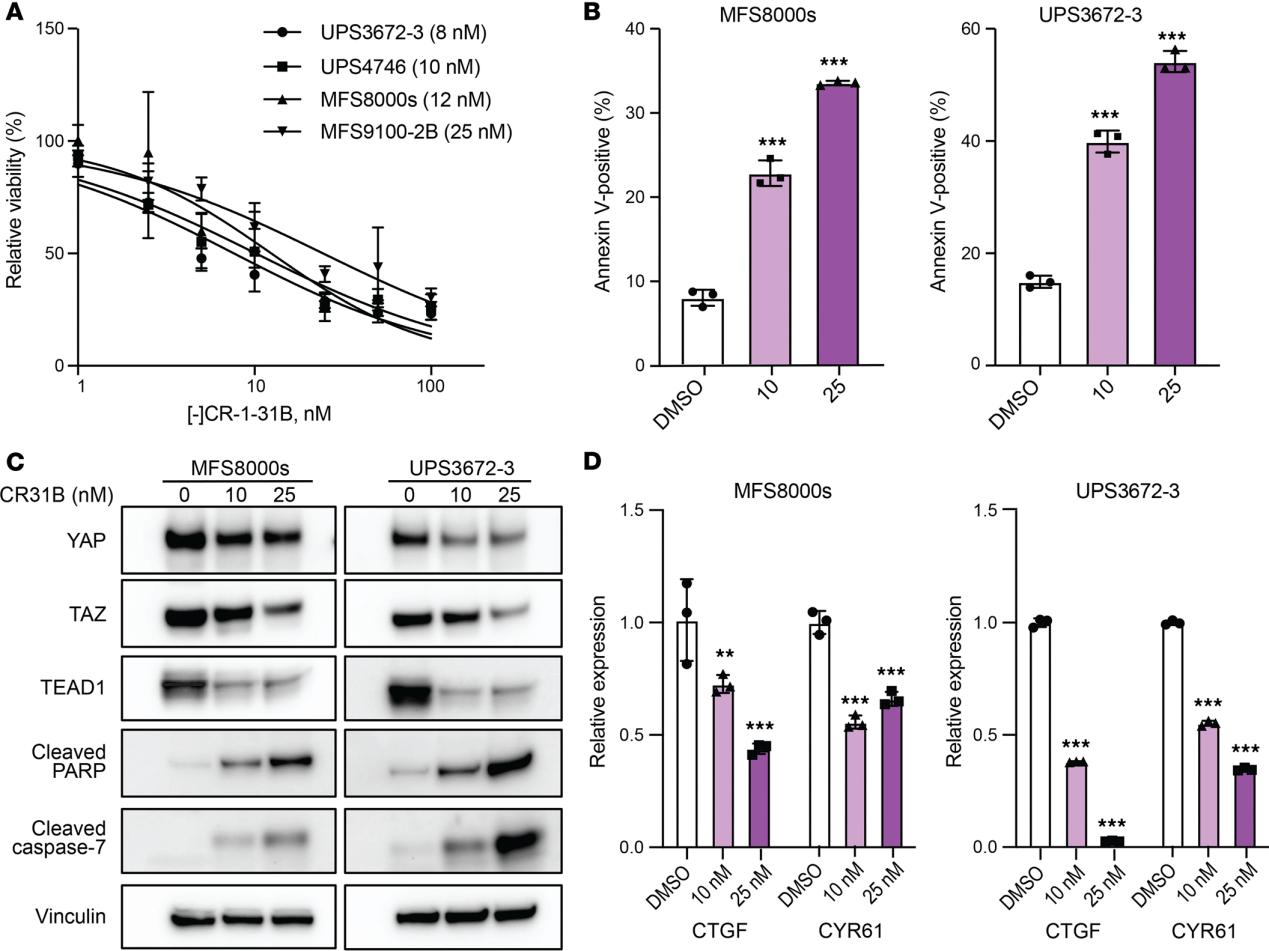
[-]CR-1-31B decreases expression of YAP, TAZ, and TEAD1 in MFS/UPS cells. (**A**) Proliferation of MFS (MFS8000s, MFS9100-2B) and UPS (UPS3673-4, UPS4746) cells after incubation with the indicated concentrations of [-]CR-1-31B for 72 hours as assessed by CyQUANT assay. (**B**) Apoptosis of MFS/UPS cells 72 hours after [-]CR-1-31B treatment as indicated by annexin V and 7-AAD costaining. (**C**) Western blots for YAP, TAZ, TEAD1, and the apoptosis markers cleaved PARP and cleaved caspase-7 in MFS/UPS cells treated with [-]CR-1-31B for 72 hours. Vinculin was used as a loading control. (**D**) mRNA expression of *CTG*F and *CYR61* in MFS/UPS cells treated with [-]CR-1-31B for 72 hours as assessed by qPCR. **P < 0.01; ***P < 0.001 by 1-way ANOVA with Dunnett’s post hoc test in **B** and 2-way ANOVA with Tukey’s post hoc test in **D**.

Turning to MFS and UPS, we found that [-]CR-1-31B was highly active against MFS (MFS8000s, MFS9100-2B) and UPS cells (UPS3672-3, UPS4746), with nanomolar IC_50_ (Figure 4A). As observed in DDLS cells, [-]CR-1-31B induced apoptosis in MFS and UPS cells (Figure 4B). Again, combined depletion of eIF4A1 and eIF4A2, validated by Western blot (Supplemental Figure 3A), significantly reduced proliferation and induced apoptosis in these cells (Supplemental Figure 3, B and C). More importantly, [-]CR-1-31B reduced expression of YAP, TAZ, and TEAD1 in MFS and UPS cells (Figure 4C). YAP and TAZ are transcriptional coactivators that interact with TEAD to promote transcription of genes required for cell proliferation and survival. [-]CR-1-31B also suppressed mRNA expression of YAP/TAZ target genes such as CTGF and CYR61 (Figure 4D). Collectively, these results indicate that eIF4A supports the proliferation and survival of DDLS, MFS, and UPS by regulating translation of common target genes despite apparent genetic complexity across these subtypes of soft tissue sarcoma.

### Copy number alterations of the Hippo pathway components in WDLS, DDLS, MFS, and UPS.

**Figure 5:**
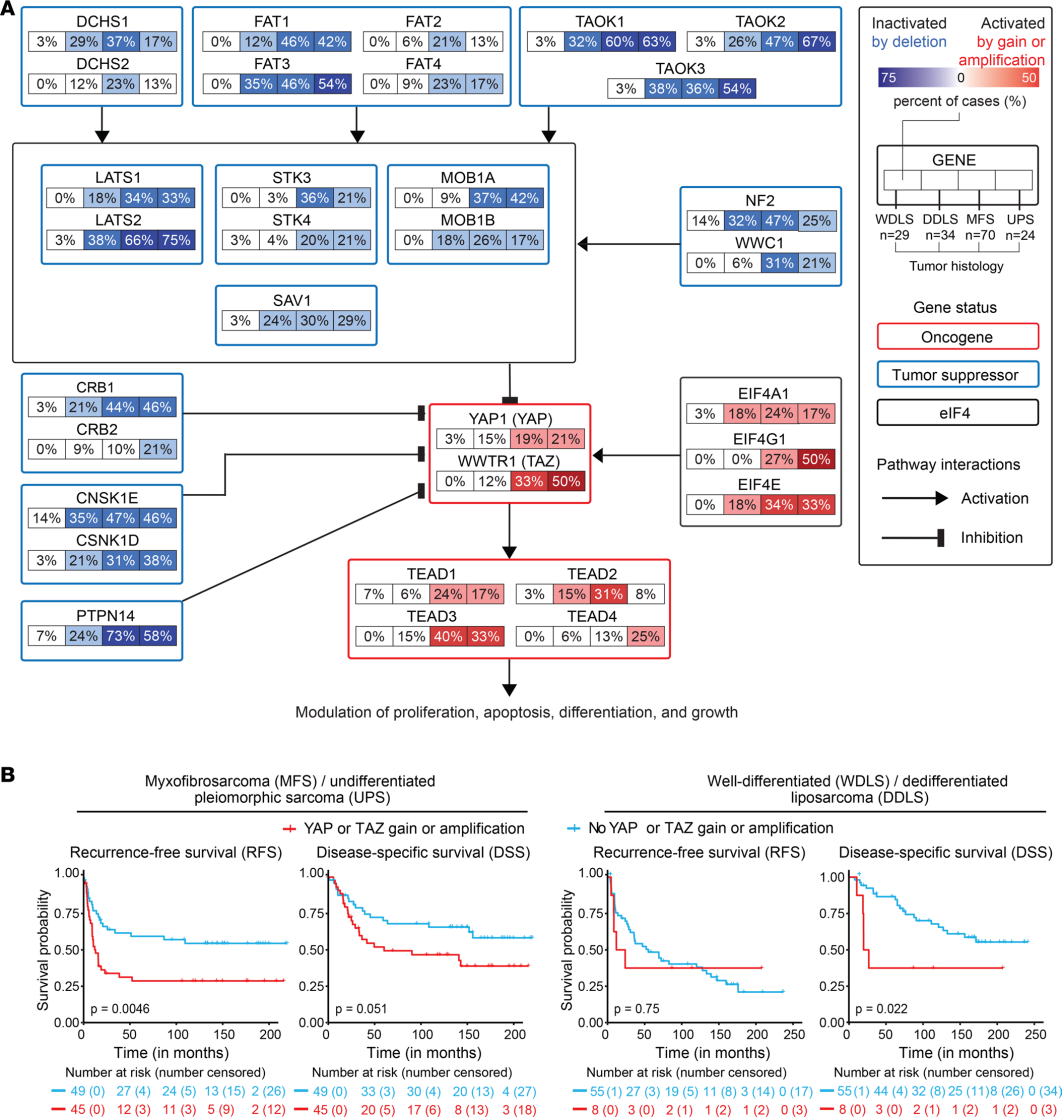
Molecular characterization of the Hippo pathway and eIF4 genes in WDLS, DDLS, MFS, and UPS tumors, and survival association of YAP/TAZ alterations. (**A**) Overall frequencies of copy number alterations (CNAs) in the Hippo pathway and eIF4 genes for WDLS, DDLS, MFS, and UPS tumors in a pathway diagram showing interactions (activation or inhibition). Only gain and amplification frequencies are shown for oncogenes and eIF4 genes and shallow and deep deletion frequencies for tumor suppressor genes. (**B**) Recurrence-free survival (RFS) and disease-specific survival (DSS) of MFS/UPS (left) and DDLS patients (right) according to the presence or absence of gain or amplification of *YAP*1 and *WWTR*1 (TAZ).

Prior studies have shown deregulation of the Hippo pathway through increased expression of the transcriptional coactivators YAP and TAZ in DDLS, MFS, and UPS (9, 23–25). To better understand the genes altered in these subtypes, we assessed copy number alterations (CNAs) in Hippo pathway genes using CGH for 107 untreated primary WDLS and DDLS and 94 MFS and UPS tumor samples (4, 37). We observed deep deletion of the Hippo pathway tumor suppressors or amplification of the Hippo pathway oncogenes in 7% of WDLS, 38% of DDLS, 37% of MFS, and 50% of UPS (Supplemental Figure 4A). Interestingly, tumor suppressor genes and oncogenes in the Hippo pathway are more frequently altered in DDLS than WDLS, suggesting that deregulation of the Hippo pathway could be involved in dedifferentiation (Figure 5A). Deep deletion of the tumor suppressors *TAOK1*, *FAT1*, and *PTPN14* was detected. At least 1 of these 3 genes was deleted in 12% of DDLS, 20% of MFS, and 13% of UPS. We also found frequent amplification or gain of the oncogenes *YAP* and *TAZ*. The combined frequency of amplification or gain in *YAP* or *TAZ* was 13% in WDLS and DDLS and 38% in MFS and UPS (Figure 5A). Amplification or gain of *YAP* and *TAZ* was significantly associated with worse recurrence-free survival (RFS) (including both local and distant recurrence) in MFS and UPS but not in WDLS and DDLS, possibly due to the small number of alterations or events in WDLS and DDLS compared with MFS and UPS (Figure 5B). Disease-specific survival (DSS) was worse in both patients with WDLS/DDLS and those with MFS/UPS with amplification or gain of *YAP* or *TAZ* (Figure 5B). On multivariable analysis, both tumor size and amplification or gain of *YAP* or *TAZ* were significantly associated with worse RFS in MFS/UPS (Supplemental Figure 4B). Together, these results indicate that the Hippo pathway transcriptional coactivators YAP and TAZ are frequently amplified or gained in DDLS, MFS, and UPS and are associated with worse clinical outcomes.

### Combined silencing of YAP/TAZ inhibits proliferation and induces apoptosis in DDLS, MFS, and UPS cells.

**Figure 6:**
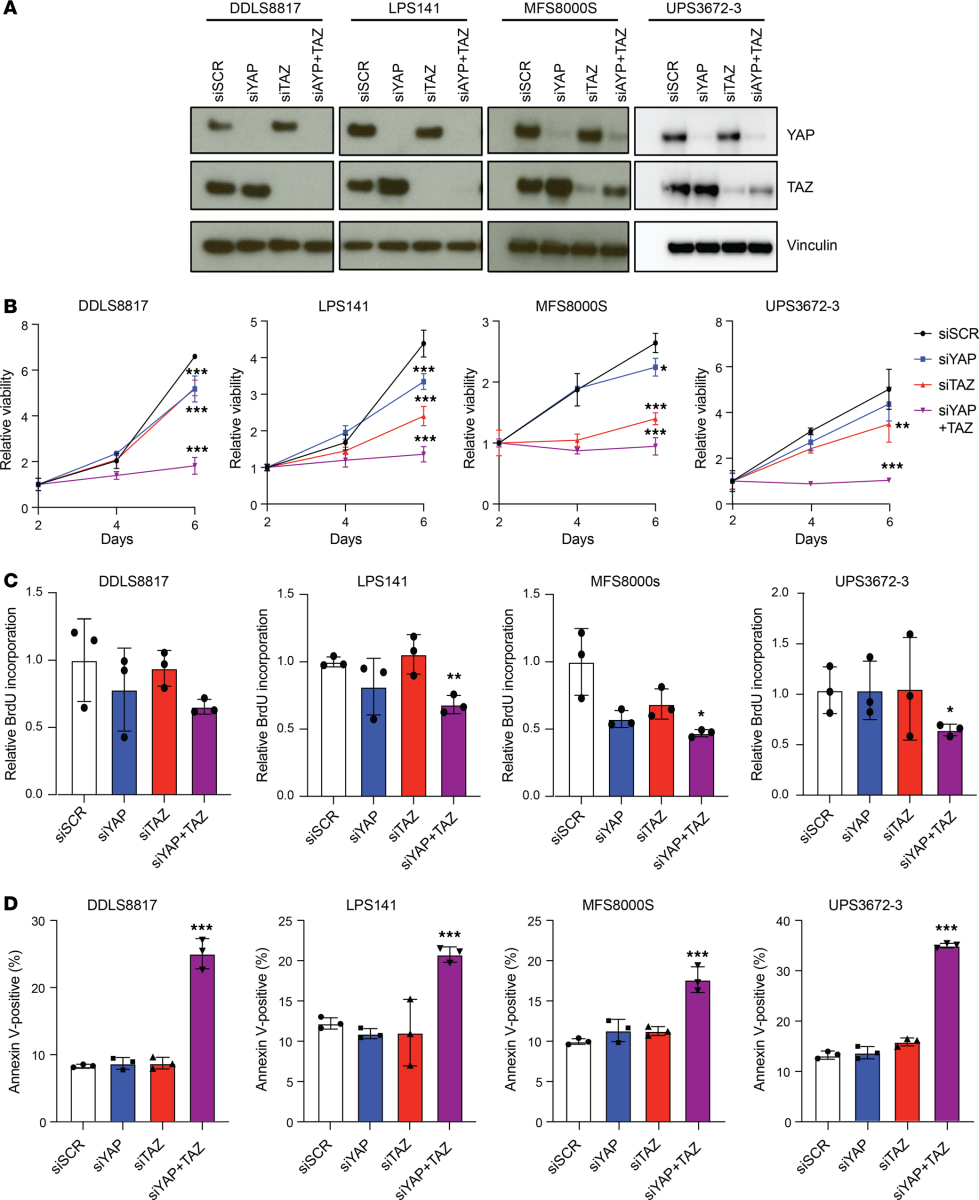
Knockdown of YAP and TAZ decreases proliferation and increases apoptosis in DDLS, MFS, and UPS. (**A**) Western blots for YAP and TAZ in siRNA-transfected cells. (**B** and **C**) Proliferation of DDLS, MFS, and UPS cells following transfection with the indicated siRNAs as assessed by CyQUANT assay and BrdU assay, respectively. (**D**) Apoptosis of DDLS, MFS, and UPS cells as measured by annexin V and 7-AAD costaining 5 days after transfection. siSCR, scramble control; siYAP, siRNA targeting YAP; siTAZ, siRNA targeting TAZ. *P < 0.05; **P < 0.01; ***P < 0.001 by 2-way ANOVA with Tukey’s post hoc test for CyQUANT in **B** and 1-way ANOVA with Dunnett’s post hoc test for BrdU in **C** and apoptosis assays in **D**.

The Hippo effectors YAP and TAZ are paralogs that share similar functions and oncogenic activation in multiple tumor types, including breast and liver cancer and various soft tissue sarcomas including DDLS, MFS, and UPS (9, 23, 24). To assess the importance of YAP and TAZ for cell survival, we knocked down YAP and TAZ using siRNA in DDLS8817, LPS141, MFS8000S, and UPS3672-3 cells. Depletion of YAP and TAZ were confirmed by Western blot (Figure 6A). Combined depletion of YAP and TAZ in DDLS8817, LPS141, MFS8000S, and UPS3672-3 further suppressed cell proliferation compared with individual depletion of YAP and TAZ (Figure 6, B and C) and increased apoptosis by 20%–30%, pointing to an essential role of YAP and TAZ in these cells (Figure 6D). These results suggest that dual inhibition of YAP and TAZ is more efficacious than inhibition of either alone in DDLS, MFS, and UPS cells.

### [-]CR-1-31B suppresses growth of DDLS, MFS, and UPS xenograft tumors.

**Figure 7:**
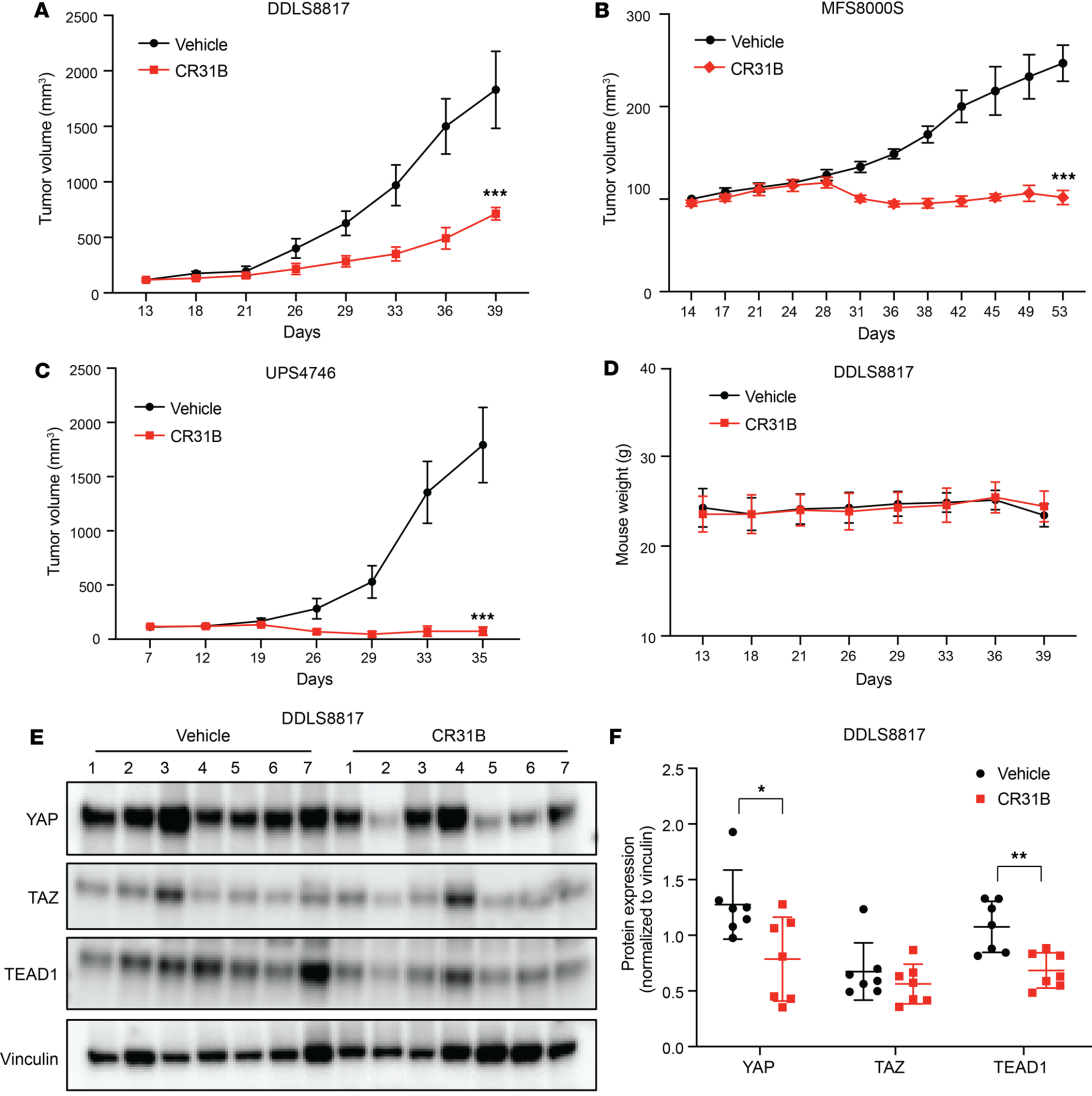
[-]CR-1-31B suppresses tumor growth of DDLS, MFS, and UPS xenografts. (**A–C**) Tumor growth DDLS8817 (**A**), MFS8000S (**B**), and UPS4746 (**C**) cell xenografts. Tumor cells were implanted s.c. into the flank of NSG mice and treated with either vehicle control or [-]CR-1-31B (0.5 mg/kg) after tumors reached ~100 mm^3^ in size. Dosing: for DDLS8817, i.v. twice a week for 4 weeks; for MFS8000S (*n* = 5 per group) and UPS4746 (*n* = 7 per group), i.p. 3 times a week for 4 weeks. Mean tumor volumes ± SD measured using calipers. (**D**) Weight of DDLS8817 xenograft-bearing mice treated with [-]CR-1-31B or vehicle. (**E**) Western blot for YAP, TAZ, and TEAD1 in DDLS8817 tumor lysates. Vinculin served as a loading control. (**F**) ImageJ quantification of YAP, TAZ, and TEAD1 protein levels normalized to vinculin (as in **C**). *P < 0.05; **P < 0.01; ***P < 0.001 by 2-way repeated measures ANOVA in **A**–**C** and unpaired 2-tailed Student’s *t* test in **F**.

To evaluate the antitumor efficacy of [-]CR-1-31B in xenograft mouse models of DDLS, MFS, and UPS, DDLS8817, MFS8000S, or UPS4746 cells were s.c. implanted into the flank of NSG mice. Treatment with [-]CR-1-31B significantly delayed tumor growth in DDLS, UPS, and MFS xenografts (Figure 7, A–C). [-]CR-1-31B treatment was well tolerated, causing no mortality, weight loss, or other signs of toxicity (Figure 7D and Supplemental Figure 5, A and B). As observed in vitro, [-]CR-1-31B led to decreased expression of YAP and TEAD1 in DDLS8817 tumors compared with vehicle treatment as observed by Western blot (*P* < 0.05) (Figure 7, E and F). Additionally, [-]CR-1-31B decreased expression of TAZ in UPS4746 and MFS8000S tumors (Supplemental Figure 5, C and D). Although the initial growth rate of MFS8000s tumors was slow over the first 28 days of the experiment, treatment with [-]CR-1-31B still significantly inhibited tumor growth compared with controls (*P* < 0.001). Notably, [-]CR-1-31B induced significant tumor regression in UPS4746 (tumor growth inhibition of 102.5%, *P* < 0.0001) (Figure 7C). However, this effect was not observed in all xenografts tested (Supplemental Figure 6A). To identify possible drivers of sensitivity and resistance, we analyzed shallow whole-genome sequencing data on these cell lines and xenografts. We found that the most sensitive xenograft models, namely UPS4743 and MFS8000s, exhibited coamplification of *EIF4G1* and *EIF4A2* (Supplemental Figure 6B). Further analysis confirmed increased mRNA expression of eIF4G1 and eIF4A2 in UPS4743 and MFS8000s, likely due to gene amplification (Supplemental Figure 6C). While eIF4G1 expression was not detected at the protein level, eIF4A2 was found to be highly expressed in UPS4746 compared with the other cell lines (Supplemental Figure 6D). Additionally, higher mRNA levels of *EIF4G1* are associated with poor RFS and DSS in patients with MFS/UPS (Supplemental Figure 6E). Together, these data demonstrate the antitumor efficacy of [-]CR-1-31B in xenograft models and show that YAP and TAZ can be targeted through eIF4A inhibition in these sarcomas in vivo.

## Discussion

In this study, we found that eIF4A inhibition shows potent activity against DDLS, MFS, and UPS despite the apparent genetic complexity across these subtypes of soft tissue sarcoma. Our results build on studies in mouse models of other cancers, including lymphoma, leukemia, breast, prostate, and pancreatic cancer, supporting the tolerability and efficacy of eIF4A inhibitors (15, 17, 20, 21, 38). A recent study also showed potent antitumor effects of eIF4A inhibition in other sarcomas, including malignant peripheral nerve sheath tumors (MPNST), Ewing sarcoma, osteosarcoma, and rhabdomyosarcoma (39).

The isoforms eIF4A1 and eIF4A2 have been shown to have different functions (40). We found that depletion of eIF4A1 augments expression of eIF4A2 and that knockdown of both isoforms induced the most extensive apoptosis in DDLS, MFS, and UPS cells. This supports the idea that increased expression of eIF4A2 may protect cells from the effects of eIF4A1 inhibition; rocaglates such as [-]CR-1-31B inhibit both isoforms, which may contribute to their efficacy (21, 34). Although [-]CR-1-31B suppressed growth of DDLS, MFS, and UPS cells, its in vivo efficacy varied among models and was strongest in the UPS4746 xenograft model, in which both *EIF4G1* and *EIF4A2* are coamplified, a frequent occurrence due to chromosomal proximity at 3q27. Elevated *EIF4G1* mRNA levels correlate with poor survival in patients with MFS and those with UPS, consistent with findings from a previous study (41). Interestingly, a genome-wide CRISPR/Cas9 screen revealed that eIF4A2 inactivation confers resistance to silvestrol, highlighting the role of eIF4A2 in modulating response to eIF4A-targeted treatments (32). MDR1/P-glycoprotein (Pgp) overexpression and NRF2 activation have also been implicated in resistance to eIF4A inhibitors (32, 42). Identification of predictive biomarkers for response to [-]CR-1-31B will be essential for further clinical development. Recently, the eIF4A inhibitor eFT226 (zotatifin) has entered phase I/II clinical trials for estrogen receptor–positive metastatic breast cancer in combination with fulvestrant and abemaciclib (43, 44).

Our ribosome profiling in the DDLS cell line identified YAP and TAZ as eIF4A-dependent mRNAs, and we confirmed that [-]CR-1-31B inhibits the expression of YAP and TAZ in DDLS, MFS, and UPS cells. eIF4A has also been shown to regulate YAP translation in pancreatic adenocarcinoma (17). Combined depletion of YAP and TAZ increased apoptosis in these sarcoma cell lines, suggesting that [-]CR-1-31B–mediated reduction of YAP and TAZ contributes to the activity of [-]CR-1-31B. Thus, we have found eIF4A inhibition as a new strategy to target otherwise undruggable oncogenes such as YAP and TAZ. The Hippo pathway has been difficult to drug, and although several companies have developed TEAD inhibitors, to date no drugs can target all 3 components of the YAP-TAZ-TEAD complex. We hypothesize that targeting both YAP and TAZ in combination is important because knockdown of either YAP or TAZ alone does not inhibit proliferation or induce apoptosis; only combined knockdown of YAP and TAZ does. This evidence supporting the importance of YAP and TAZ in driving WD/DDLS, MFS, and UPS is bolstered by prior studies showing that Hippo pathway deregulation promotes tumorigenesis in these sarcoma types through activation of YAP and TAZ (23, 24). Overactivity of YAP and TAZ has also been implicated in the formation of embryonal and alveolar rhabdomyosarcoma, myxoid liposarcoma, fibrosarcoma, leiomyosarcoma, and synovial sarcoma (23, 45–47). This and other studies suggest that targeting the translation of the YAP/TAZ-TEAD mRNAs could be a promising novel approach in sarcoma.

A multiplatform molecular characterization of the Hippo pathway across 33 cancer types showed that TAOK1 on 17p (distinct from p53) is frequently deleted in sarcoma, but the study did not analyze Hippo pathway CNAs in specific sarcoma types (48). Analyzing our own copy number data, we found frequent deletion in the Hippo pathway tumor suppressors *TAOK1*, *FAT1*, and *PTPN14*. TAOK1, a direct kinase for MST1/2 and LATS1/2, is critical for LATS1/2 activation, leading to inhibition of YAP oncogenic function via cytoplasmic sequestration (49). FAT1 interacts with Hippo signaling components such as NF2, MST1/2, and LATS1/2, leading to inactivation of YAP (50). Similarly, PTPN14 can inhibit YAP/TAZ activity through direct interaction or interaction with upstream regulators LATS1/2 (51–53). Thus, loss of TAOK1, FAT1, or PTPN14 may increase YAP/TAZ nuclear localization and expression of their target genes. We also found frequent amplification or gain of YAP and TAZ that was associated with poor RFS in MFS/UPS, independently from clinical features such as tumor size and subtype. The higher frequency of gain or amplification of YAP/TAZ in DDLS (21%) than WDLS (3%) suggests that this event may contribute to dedifferentiation, as these genes are reported to inhibit adipocyte differentiation (30, 31, 54). More comprehensive study is needed to fully understand how these genetic alterations in the Hippo pathway affect the development and differentiation state of sarcoma.

In summary, our results identify the eIF4A RNA helicase as a potential common drug target for DDLS, MFS, and UPS, for which effective systemic treatments are not currently available. eIF4A inhibition represents an alternative therapeutic strategy to target undruggable oncogenes such as YAP and TAZ. Since [-]CR-1-31B was well tolerated, these promising results support further evaluation of [-]CR-1-31B or related eIF4A inhibitors for advanced or unresectable DDLS, MFS, and UPS in early phase clinical trials.

## Methods

### Sex as a biological variable.

Sex was not considered as a biological variable in this study. All mice used were female NSG mice.

### Chemicals.

[-]CR-1-31B was purchased from WuXi AppTec and suspended in DMSO for *in vitro* experiments and in 10% captisol (Sigma-Aldrich) in sterile water for in vivo experiments. Cycloheximide (C7698) and MG-132 (M7449) were purchased from Sigma.

### Cell culture.

All WD/DDLS and MFS/UPS cell lines were previously established from fresh human tumor samples under IRB-approved protocols and validated as described previously (4, 55). Cells were grown in a 1:1 mixture of high-glucose DMEM and F12 medium with 10% FBS, 2 mM L-glutamine, 100 units/mL penicillin, and 100 μg/mL streptomycin, and maintained in a 37°C incubator with 5% CO_2_. All cell lines were confirmed as negative for *Mycoplasma* prior to use in assays.

### Cell proliferation, colony formation, and apoptosis assays.

Cells were seeded at 2,000 cells per well in 96-well plates for cell proliferation assays and 5,000 or 100,000 cells per well in 6-well plates for colony formation and apoptosis assays, respectively. Cell proliferation was assessed by estimating DNA content using the CyQUANT Cell Proliferation Assay Kit (Thermo Fisher Scientific). Colonies were detected by staining with crystal violet. BrdU incorporation was assessed using BrdU Cell Proliferation Kit (Cell Signaling Technology). Apoptosis was evaluated by measuring costaining for Annexin V-PE and 7-aminoactinomycin D (7-AAD) using the Guava Nexin Assay reagent (Luminex) following the manufacturer’s instructions on the Guava EasyCyte flow cytometer (Luminex).

### Ribosome footprinting.

Human DDLS DDLS8817 cells were treated with DMSO or [-]CR-1-31B (10 nM) for 2 hours followed by cycloheximide for 10 minutes. Total RNA and ribosome-protected fragments were isolated, ligated, and reverse-transcribed following published protocol (56). Deep-sequencing libraries were generated from these fragments by PCR amplification and sequenced on the HiSeq 2000 platform.

Sequence alignment was carried out as described in previous studies (15, 17). Briefly, RF reads were filtered based on quality score (≥ 75% of nucleotides with score ≥ 25) trimmed to remove the 3′ linker sequence (5′-CTGTAGGCACCATCAAT-3′), and reads shorter than 15 nucleotides after linker trimming were removed using FASTX-Toolkit (http://hannonlab.cshl.edu/fastx_toolkit/index.html). Ribosomal RNA was removed by alignment of reads to the ribosome RNA sequences of the GRCh37 from the USCS Table Browser (https://genome.ucsc.edu/cgi-bin/hgTables). Remaining RF reads were mapped to the human genome sequence GRCh37 using HISAT2 with default parameters. Only uniquely aligned reads were further analyzed. Total mRNA-seq reads were aligned similarly with HISAT2 using splice-aware alignment, keeping only uniquely aligned reads. Aligned reads for both RF and mRNA-seq were quantified using featureCounts, with protein-coding gene annotations from GRCh37 as input. Only reads aligned to the exonic regions of protein-coding genes were used for downstream analysis. TE was quantified from ribosome footprinting and RNA-seq data using Ribo-diff (57). Genes with ≥ 10 normalized read counts in the sum of RF and RNA-seq data were used as input, which resulted in 17,930 genes in total. Genes with significantly changed TE were defined as those with *q* < 0.01. Ribosomal distribution curves for each gene were plotted as described previously (15).

### Motif analysis.

Motifs were predicted within the 5′-UTR sequence of the longest transcript for each gene with significantly increased or decreased TE using DREME (58). The occurrence of significant motifs (E < 0.05 and *P* < 1 × 10^–8^) were called using FIMO (59) with default parameters for strand-specific prediction of all 5′-UTR sequences.

### Cloning of the dual-luciferase constructs.

5′-UTRs were cloned into a dual-luciferase reporter system (pFR_HCV_xb plasmid), which contains an HSV-TK promoter, firefly luciferase gene, HCV IRES, and Renilla luciferase gene. Each 5′-UTR (β-globin [NM_000518], negative control: 50 bp, 44% GC; c-MYC [NM_002467], positive control: 363 bp, 65% GC; TAZ [NM_015472], TAZ-WT: 264 bp, 69% GC; TAZ-mutant: 264 bp, 65% GC) was cloned downstream of the HSV-TK promoter and positioned immediately before the firefly luciferase AUG start codon using the NEB HiFi DNA assembly method. Cloning strategies were designed using SnapGene software; sequences are available upon request.

### Dual-luciferase reporter assay.

The day before transfection, 50,000 DDLS8817 cells per well were seeded in a 24-well plate in 1 mL DMEM-F12 supplemented with 10% FBS, incubated at 37°C, 5% CO_2_. Cells were transfected using Jetprime reagent according to the manufacturer’s instructions 4–6 hours before treatment with 10 nM CR31B or DMSO in fresh medium for 24 hours at 37°C at 5% CO_2_. The Dual-Luciferase Reporter Assay (Promega) was then performed according to the manufacturer’s instructions, and luminescence was measured using a Synergy plate reader. Luminescence values of firefly luciferase were normalized to those of renilla luciferase before calculating the percentage of relative expression of firefly luciferase.

The TAZ-WT 5′-UTR sequence is as follows (GQ-forming sequences are in bold): AGTCCGGGAGCTGCTGCGGCCGCGCTGTCTGC-TTCTCCTGCGCCTCCTTTTCGCCCAGCACTAGCGCCTTAGGCCAGCTCGGGGGATGTGAGAGCCGAAGCCCTTAGACTGCCAGGCACA-GAGTCGGGTCGGGATTTGTCAGCCAAGCCTCGGCTCCAGCTCCGCAATCTCGGGACTCACCCGAGCGACCCAGGCCCGACGGCAAGTT-**CGGGCGGGACGGCGGCCGCCGCGCGC**TCAGGCTCAGCTTCGCTGCCCGCCCAGAAG. The TAZ-mutant 5′-UTR sequence is as follows (the mutant GQ sequence is in bold): AGTCCGGGAGCTGCTGCGGCCGCGCTGTCTGCTTCTCCTGCG-CCTCCTTTTCGCCCAGCACTAGCGCCTTAGGCCAGCTCGGGGGATGTGAGAGCCGAAGCCCTTAGACTGCCAGGCACAGAGTCGGGTCGGG-ATTTGTCAGCCAAGCCTCGGCTCCAGCTCCGCAATCTCGGGACTCACCCGAGCGACCCAGGCCCGACGGCAAGTT**GACAACGTCAGC-GTTCAGCGTTCCAA**TCAGGCTCAGCTTCGCTGCCCGCCCAGAAG.

### siRNA transfection.

For transient knockdown, cells were transfected with either nontargeting pool small interfering RNA (siRNA) (D-001810-10-0005) or siRNAs targeting human *eIF4A1* (L-020178-00-0005), *eIF4A2* (L-013758-01-0005), *YAP1* (L-012200-00-0005), or *WWTR1* (L-016083-00-0005) (ON-TARGETplus Smart Pool, Horizon) using DharmaFECT1 according to the manufacturer’s recommendations (Horizon).

### Western blotting.

Cells were lysed in 1% SDS lysis buffer (1% SDS, 50 mM Tris-HCl pH 8.0, 10 mM EDTA pH 8.0, 10% glycerol) and immediately boiled to denature proteins. Xenograft tumor tissues were lysed in SDS lysis buffer. In total, 20–30 μg of protein was electrophoretically separated on 4%–12% gradient Bis-Tris gels (Thermo Fisher Scientific), before being transferred to PVDF membranes (Immobilon, EMD Millipore). Membranes were incubated overnight with primary antibodies: cleaved PARP (Cell Signaling Technology, 9541), cleaved caspase-7 (Cell Signaling Technology, 9491), vinculin (Sigma, V4505), eIF4A1 (Cell Signaling Technology, 2490), eIF4A2 (Abcam, 31218), YAP (Cell Signaling Technology, 12395), TAZ (Cell Signaling Technology, 8418), or TEAD1 (Cell Signaling Technology, 12292). Proteins were detected by incubation with secondary HRP-conjugated anti-rabbit IgG (Cell Signaling Technology, 7074) or anti-mouse IgG (Cell Signaling Technology, 7076), followed by washing and addition of SuperSignal West Pico or Fempto Chemiluminescent substrate (Thermo Scientific). Western blot images were quantified using ImageJ software (NIH).

### Real-time PCR.

Total RNA was extracted using the RNeasy Mini kit (Qiagen 74104). cDNA was synthesized using SuperScript III First Strand Synthesis System (Thermo Fisher Scientific). qPCR using TaqMan Gene Expression Assays (Applied Biosystems) was done on the Viia 7 Real-Time PCR System (Thermo Fisher Scientific). Relative expression was quantified as ΔΔCT, with 18S rRNA as an endogenous control. Primers were as follows: 18s rRNA (Hs99999901_s1), YAP1 (Hs00902711_g1), WWTR1 (Hs00210007_m1), TEAD1 (Hs00173359_m1), CTGF (Hs00170014_m1), CYR61 (Hs00155479_m1), eIF4A2 (Hs00756996_g1), and eIF4G1 (Hs00191933_m1).

### Retroviral transduction.

pBABE-puro was a gift from Hartmut Land (University of Rochester), Jay Morgenstern (Warp Drive Bio), and Robert Weinberg (Massachusetts Institute of Technology) (Addgene plasmid #1764) (60), pBabe-puro YAP1 was a gift from Joan Brugge (Harvard University) (Addgene plasmid #15682) (61), and pQCXIH-TAZ was a gift from Kunliang Guan (UCSD, San Diego, California, USA) (Addgene plasmid #32841) (62). To generate viral supernatant, the pBABE-puro or pQCXIH-Taz plasmids along with helper plasmids gag-pol and VSVG were transfected into HEK293FT cells (Invitrogen) using lipofectamine 2000 (Invitrogen). Cells were infected using complete medium containing viral supernatant and 10 mg/mL polybrene (Sigma-Aldrich) and selected using 2 μg/mL puromycin (Sigma-Aldrich) or 1 mg/mL hygromycin (Invitrogen).

### Selection of genes involved in the Hippo pathway.

The Hippo pathway gene set was separated into oncogenes or tumor suppressors as previously categorized (7).

### GC normalization and random allelic expression (RAE) analysis of array comparative genomic hybridization (CGH) data.

Analysis of array CGH data used a customized normalization method that was developed to account for the fact that signals of CGH probes targeting adjacent regions in the genome are highly correlated with one another and all probes’ signals are correlated with target GC content. Complete methods and code are available at https://github.com/soccin/CGHPipe (commit ID 94f8980) (63, 64). To identify CNAs, the normalized dataset was processed using a custom pipeline consisting of standard circular binary segmentation using R/Bioconductor DNAcopy followed by processing using GISTIC (v2) (65) to generate gene-level copy number calls.

### Survival analysis.

RFS was calculated from the date of surgical resection to the date of the first local or distant recurrence event. DSS was calculated from the date of surgical resection to the date of death from sarcoma. The effect of gain or amplification of *YAP1* (YAP) or *WWTR1* (TAZ) on DSS and RFS was evaluated using Kaplan-Meier and multivariable analyses adjusted for tumor histology and tumor size using the survminer R package available on GitHub (https://github.com/kassambara/survminer; commit ID cc8abfc).

### Animal studies.

For xenograft models, serially transplanted DDLS8817, MFS8000s, or UPS4746 tumors were s.c. implanted into the flank of female NSG mice. Treatment began when tumors reached 100–150 mm^3^. DDLS8817 tumor-bearing mice were dosed i.v. with vehicle or [-]CR-1-31B (0.5 mg/kg, twice per week), and MFS8000s or UPS4746 tumor-bearing mice were dosed i.p. with vehicle or [-]CR-1-31B (0.5 mg/kg, 3 times per week) for 3–4 weeks. Tumor growth was measured twice per week. Toxicity was monitored by measuring weight, and clinical observation was conducted daily until the end of the experiment.

### Statistics.

Experimental data were analyzed using GraphPad Prism version 9.0. The significance of differences was tested by 1-way ANOVA except for analysis of protein expression in Western blots, which used unpaired 2-tailed Student *t* tests, and differences in tumor growth and mouse weight, which used 2-way repeated measures ANOVA. Data are shown as mean ± SD. The significance of motif enrichment was calculated in the DREME program using the Fisher exact test. mRNA expression was compared between groups using the Wilcoxon test.

### Study approval.

All research involving human biospecimens and data in this study was approved by MSK’s IRB. All patients from whom biospecimens and/or genomic data were analyzed provided written informed consent to broad research use of such material and/or information. Mouse studies were approved by MSK’s IACUC.

### Data availability.

Array CGH expression data are publicly available via cBioPortal study: https://www.cbioportal.org/study/summary?id=sarcoma_msk_2026 Ribosome profiling data are available from GEO (accession no. GSE227676). Supporting Data Values are available in the linked file online.

## Author contributions

YMK, HGW, and SS conceptualized the study and selected and refined methodology. YMK, PM, US, RMDQ, KC, NP, TO, and AK performed the experiments. YMK, PM, ES, US, RMDQ, NL, S Smith, JL, BJ, and NDS curated data. YMK, PM, ES, US, RMDQ, TO, JL, NL, S Smith, and NDS analyzed data. YMK, ES, JL, US, and RMDQ created the graphical displays of results. ZO, HGW, and S Singer acquired funding and supervised the study. YMK wrote the original draft of the manuscript. YMK, PM, ES, US, TO, NDS, HGW, and S Singer reviewed and edited the manuscript. The order of co–first authors reflects their relative contributions to primary data collection and initial manuscript drafting.

## Conflict of interest

NDS is an owner of and holds equity interests in Solvuu LLC (no associated compensation).

## Funding support

Supported by grants from the following organizations:

- NIH grant P50 CA217694 (to S Singer and HGW).
- NIH grant R35 CA252982 (to HGW).
- Cancer Center Support Grant P30 CA008748.

## Supplementary Material

Supplemental data

Unedited blot and gel images

Supplemental table 1

Supplemental table 2

Supporting data values

## Version 1 04/07/2026

In-Press Preview

## Version 2 05/22/2026

Electronic publication
