# Supplementary material for: Targeting eIF4A-dependent translation in genetically complex sarcoma
Source: JCI Insight. 2026 Apr 7;11(10):e192936. doi: 10.1172/jci.insight.192936 (PMC13232726; doi:10.1172/jci.insight.192936)
Supplement: Supplemental data [file jciinsight-11-192936-s260.pdf]

Supplemental figures for Kim et al., Targeting eIF4A-dependent translation in genetically complex sarcoma

SFigure 1. Knockdown of eIF4A inhibits proliferation and induces apoptosis in DDLS cells. **A**, Western blots for eIF4A1 and eIF4A2 in protein lysates prepared from siRNA-transfected cells. **B**, Proliferation of DDLS8817 and LPS141 cells transfected with the indicated siRNAs as assessed by CyQUANTassay. **C**, Proliferation of DDLS8817 and LPS141 cells measured by BrdU staining 3 days post-transfection. **D**, Apoptosis of DDLS8817 and LPS141 cells as measured by Annexin V and 7-AAD costaining 5 days after transfection. siSCR, scramble control; si4A1, siRNA targeting eIF4A1;si4A2, siRNA targeting eIF4A2. *, p < 0.05; **, p < 0.01; ***, p < 0.001.

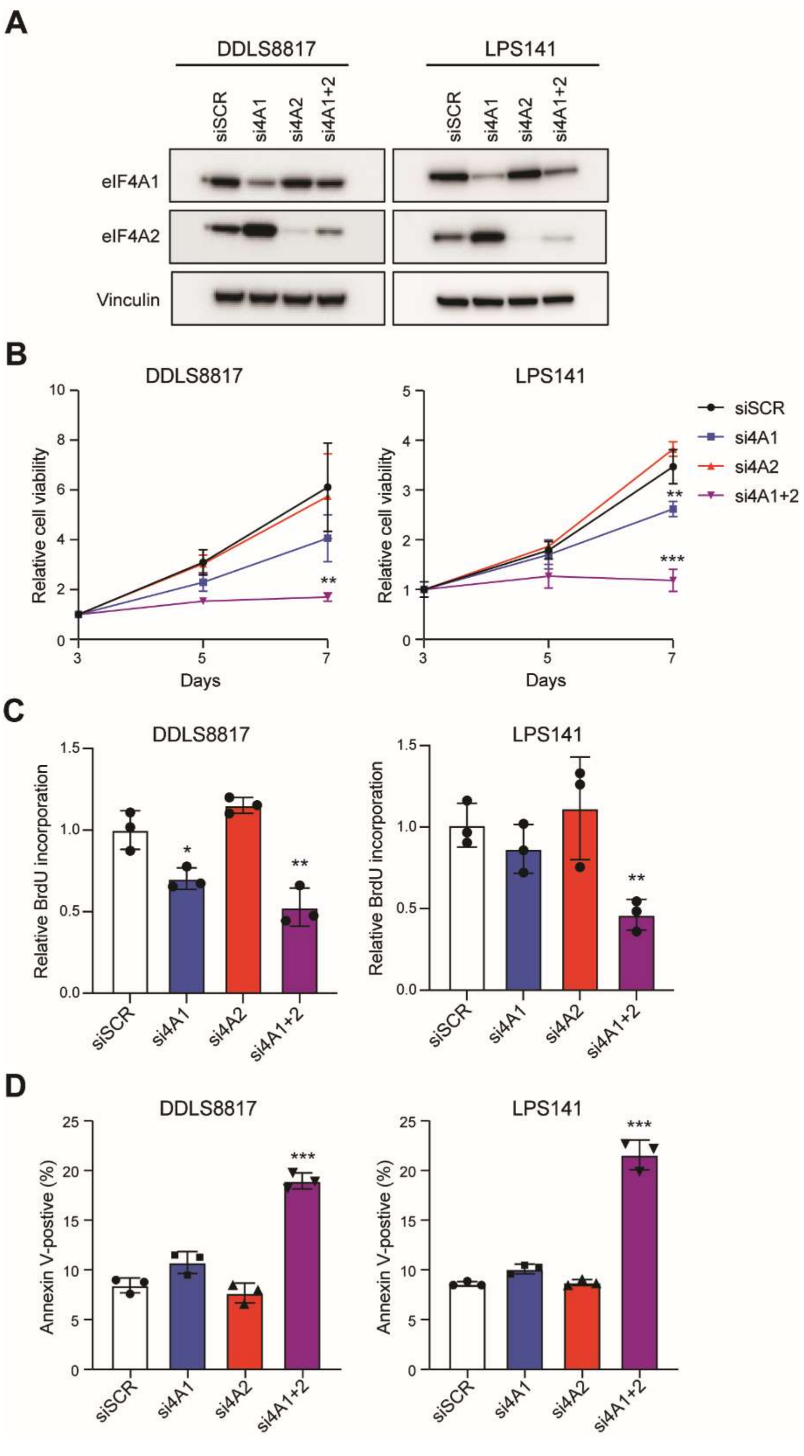

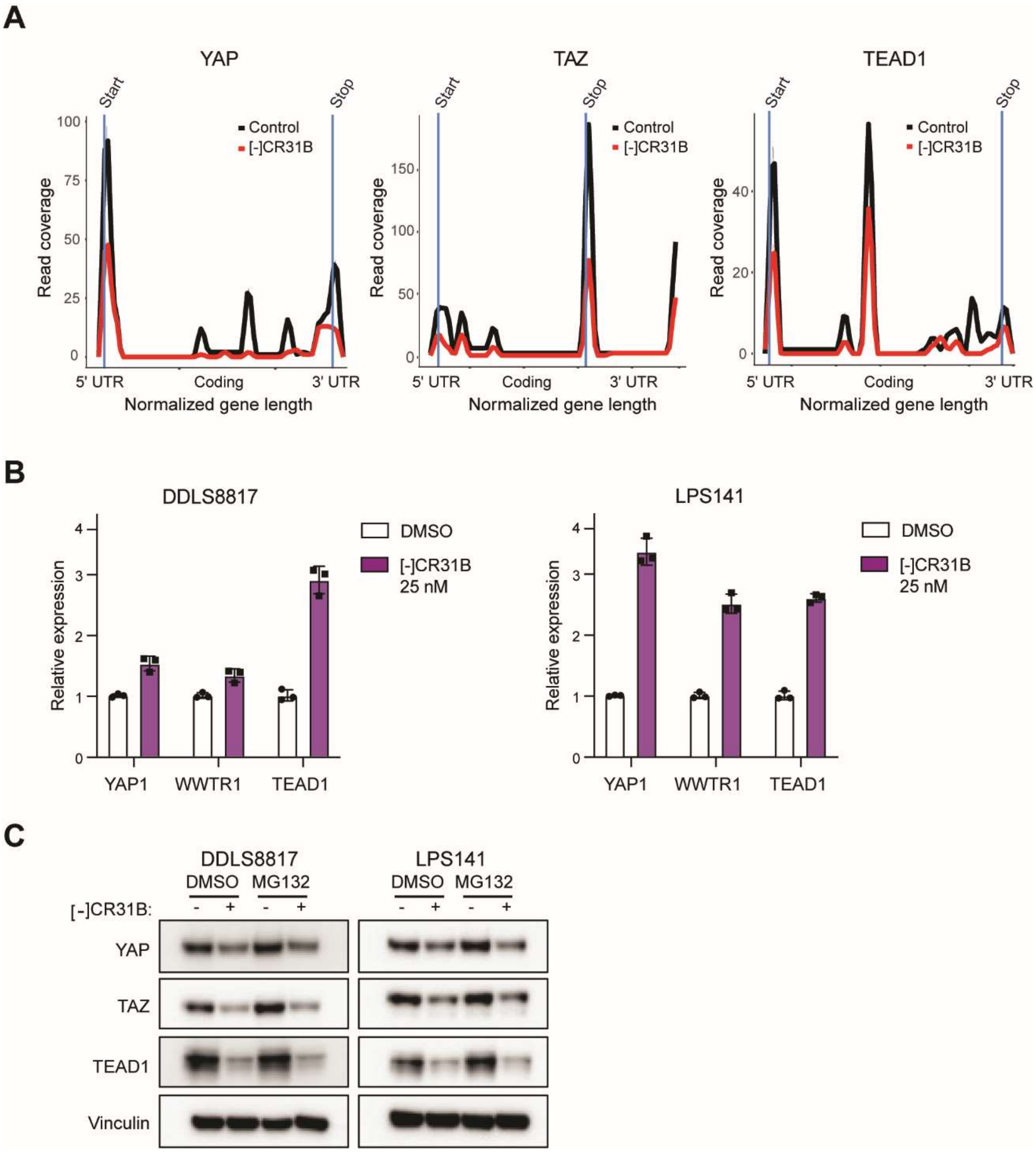

SFigure 2. [-]CR-1-31B reduces translation of YAP, TAZ, and TEAD1. **A**, Effect of [-]CR-1-31B on the distribution of ribosomal footprints on the YAP1, WWTR1 (encoding TAZ), and TEAD1 genes. Gene lengths are normalized. **B**, mRNA expression of YAP1, WWTR1, and TEAD1 in DDLS8817 and LPS141 cells treated with DMSO or 25 nM [-]CR-1-31B for 72 h as assessed by qRT-PCR. **C**, Western blot for YAP, TAZ, and TEAD1 in DDLS8817 and LPS141 cells treated with [-]CR-1-31B for 24 h, followed by 10 μM MG-132 or control for 6 h.

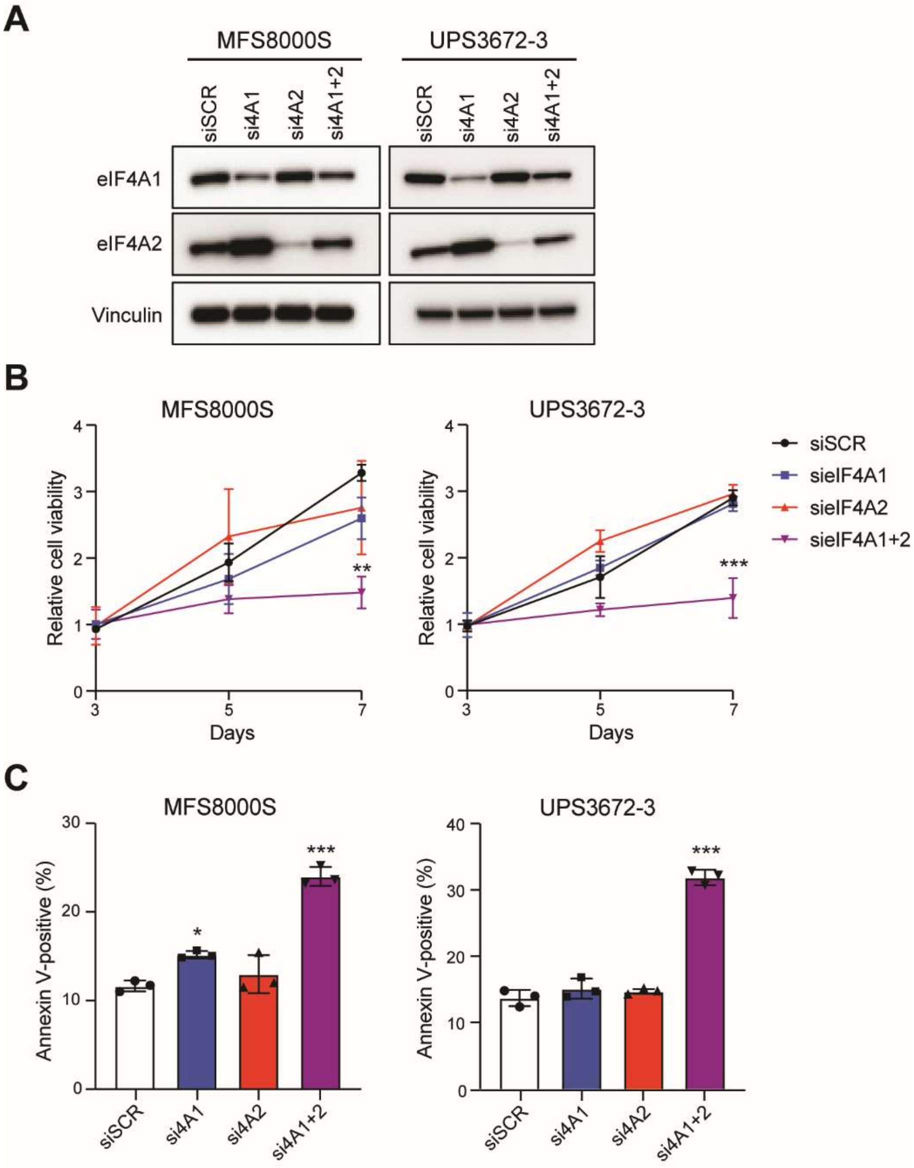

SFigure 3. Knockdown of eIF4A inhibits proliferation and induces apoptosis in MFS and UPS cells. **A**, Western blots of protein lysates prepared from siRNA transfected cells and probed for indicated proteins. **B**, Proliferation of MFS8000S and UPS3672-3 cells transfected with the indicated siRNAs as assessed by CyQUANT assay. **C**, Apoptosis as measured by Annexin V and 7-AAD co-staining 7 days after transfection. siSCR, scramble control; si4A1, siRNA targeting eIF4A1; si4A2, siRNA targeting eIF4A2. *, $p < 0.05$; **. $p < 0.01$; ***, $p < 0.001$.

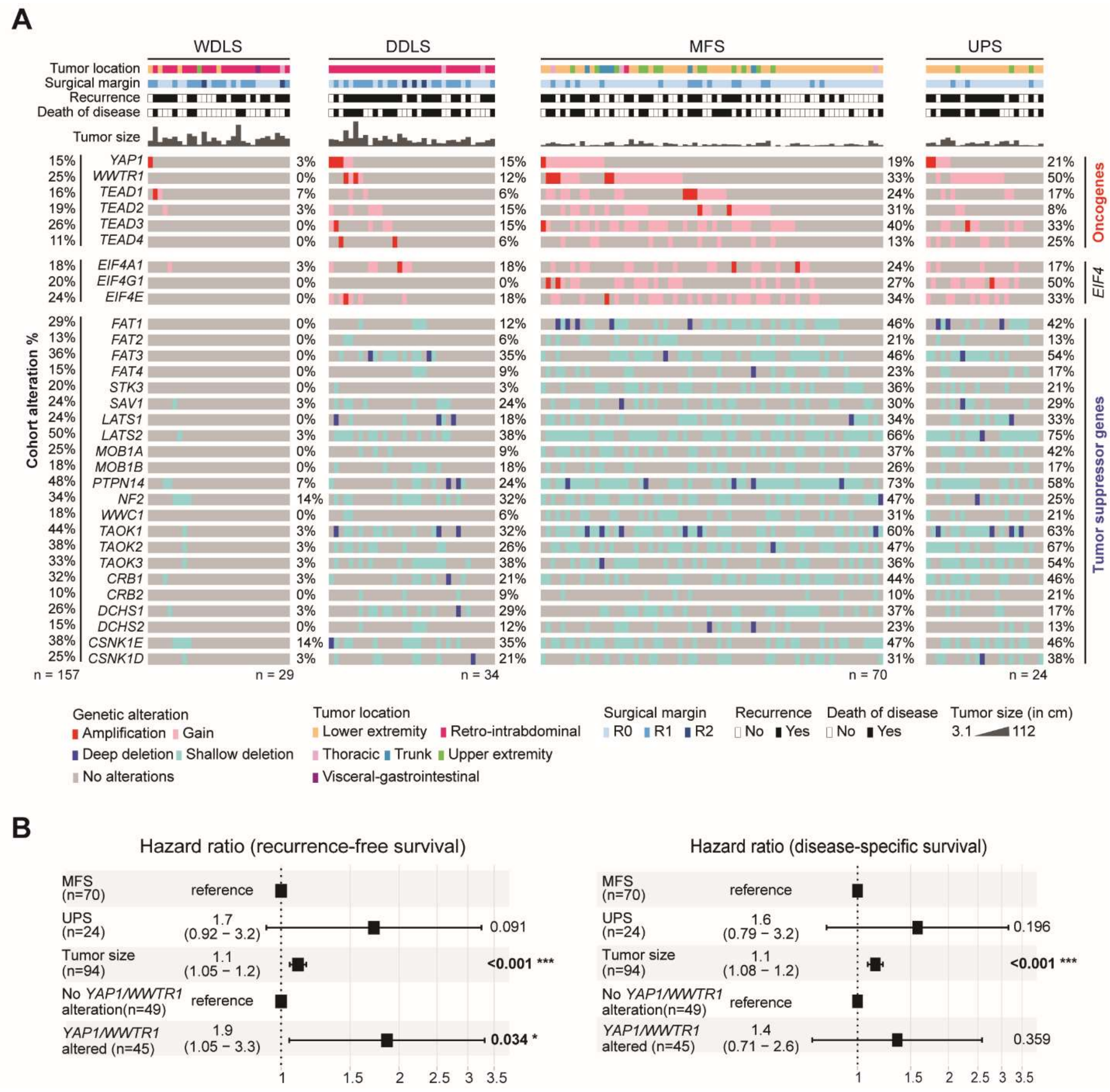

SFigure 4. **A**, Oncoprint illustrating overall copy number alteration (CNA) frequencies in Hippo pathway genes and eIF4 genes among WDLS, DDLS, MFS, and UPS tumors. Only gains and amplifications are shown for oncogenes and shallow and deep deletions for tumor suppressor genes. **B**, Multivariable hazard ratios (HRs) and associated p-values for recurrence-free (left) and disease-specific (right) survival according to indicated variables. HRs for gain or amplification of YAP1 (YAP) or WWTR1 (TAZ) calculated after adjustment for tumor subtype (MFS vs. UPS) and tumor size (as a continuous variable).

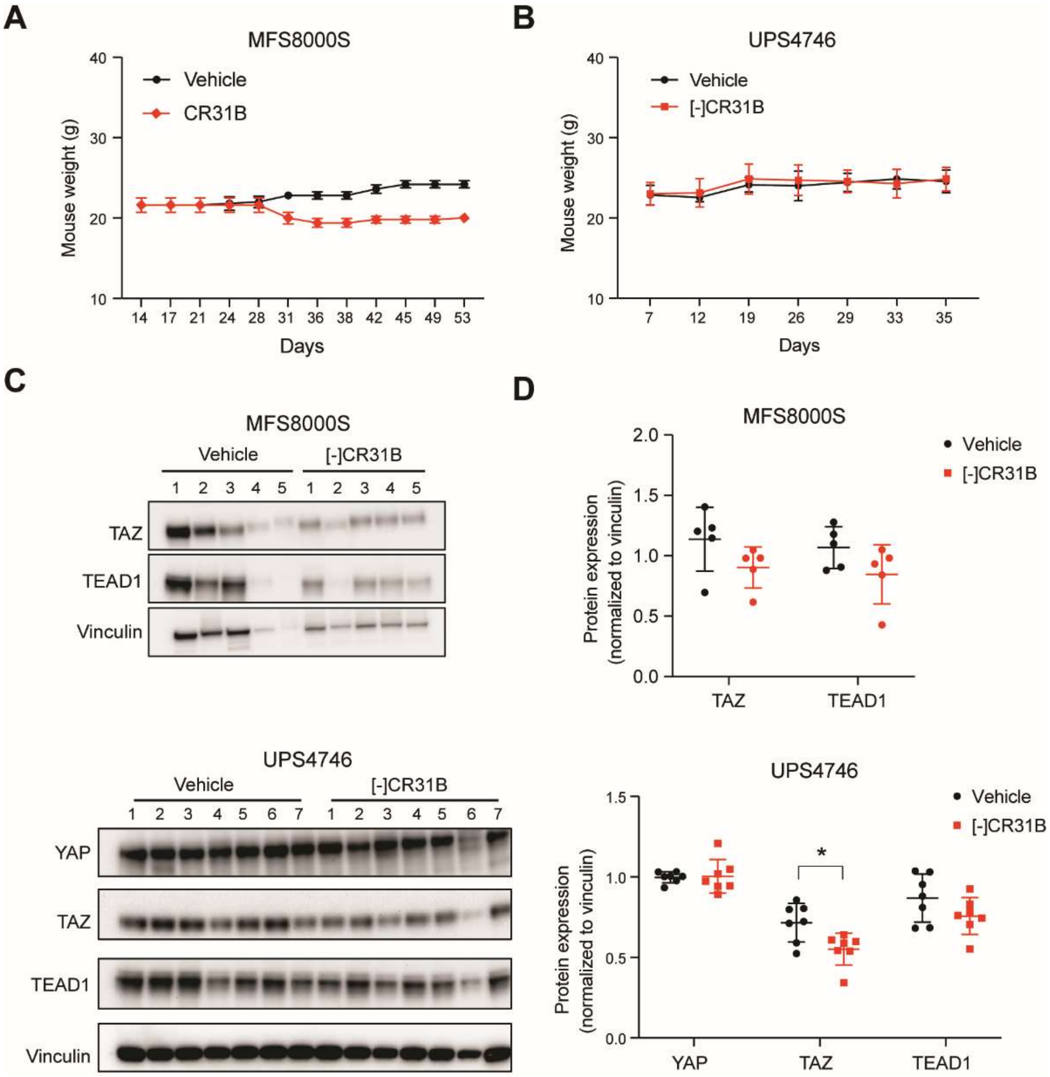

SFigure 5. In vivo safety and efficacy of the eIF4A inhibitor [-]CR-1-31B in MFS and UPS models. A–B, Weight of (**A**) MFS8000S and (**B**) UPS4746 xenograft-bearing mice during [-]CR-1-31B treatment. **C**, Western blots for YAP, TAZ, and TEAD1 in MFS8000S and UPS4746 tumor lysates. Vinculin served as a loading control. **D**, ImageJ quantification of YAP, TAZ, and TEAD1 protein expression in (**C**) normalized to vinculin. *, $p < 0.05$.

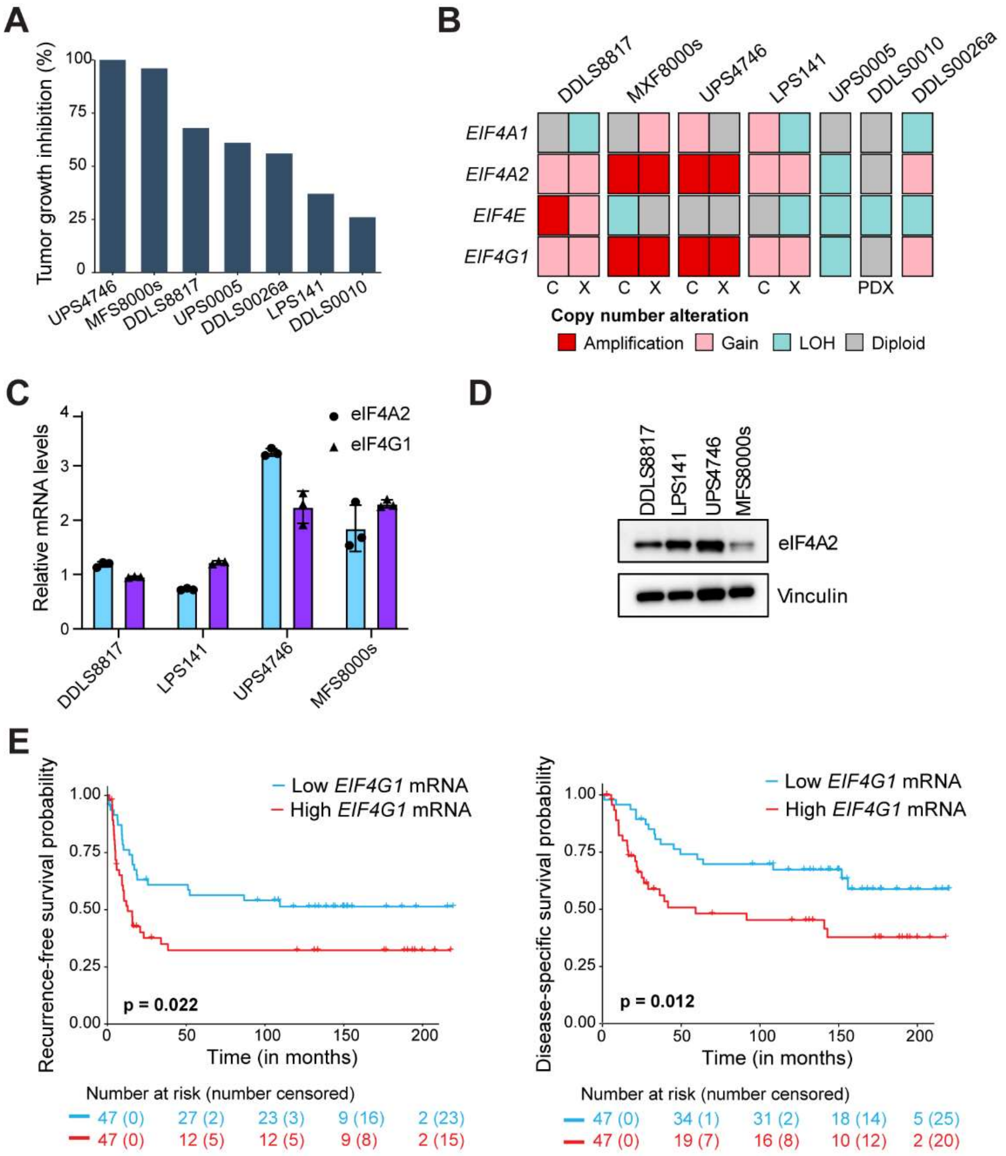

SFigure 6. [-]CR-1-31B shows stronger in vivo efficacy in eIF4G1 and eIF4A2 co-amplifiedmodels compared to non-amplified DDLS, MFS, and UPS models. **A**, Tumor growth inhibition (%) in DDLS, MFS, and UPS xenograft models treated with [-]CR-1-31B. **B**, Oncoprint illustrating copy number alterations in eIF4 genes across DDLS, MFS, and UPS cell lines (C), their corresponding cell line-derived xenografts (X), and PDXs. LOH, loss of heterozygosity. **C**, mRNA expression levels of eIF4A2 and eIF4G1 in DDLS8817, LPS141, MFS8000s and UPS4746 relative to control L090310 cells using qRT-PCR. **D**, Western blot for eIF4G2 in DDLS8817, LPS141, UPS4746, and MFS8000s. **E**, Recurrence-free survival (RFS) and disease-specific survival (DSS) ofMFS/UPS patients according to mRNA expression of eIF4G1.
