# Supplementary material for: Targeting eIF4A-dependent translation in genetically complex sarcoma
Source: JCI Insight. 2026 Apr 7;11(10):e192936. doi: 10.1172/jci.insight.192936 (PMC13232726; doi:10.1172/jci.insight.192936)

Figure 1D

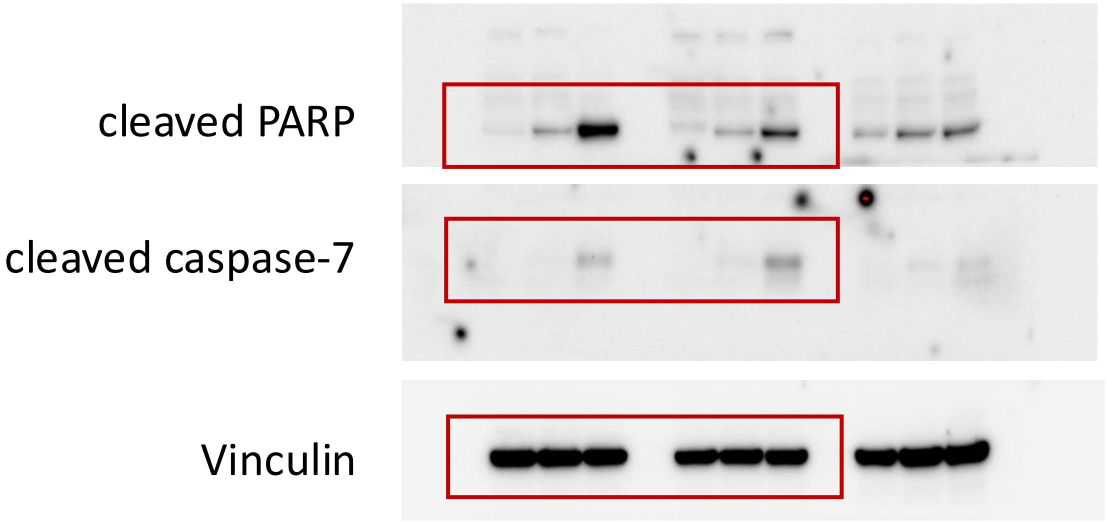

Figure 2D

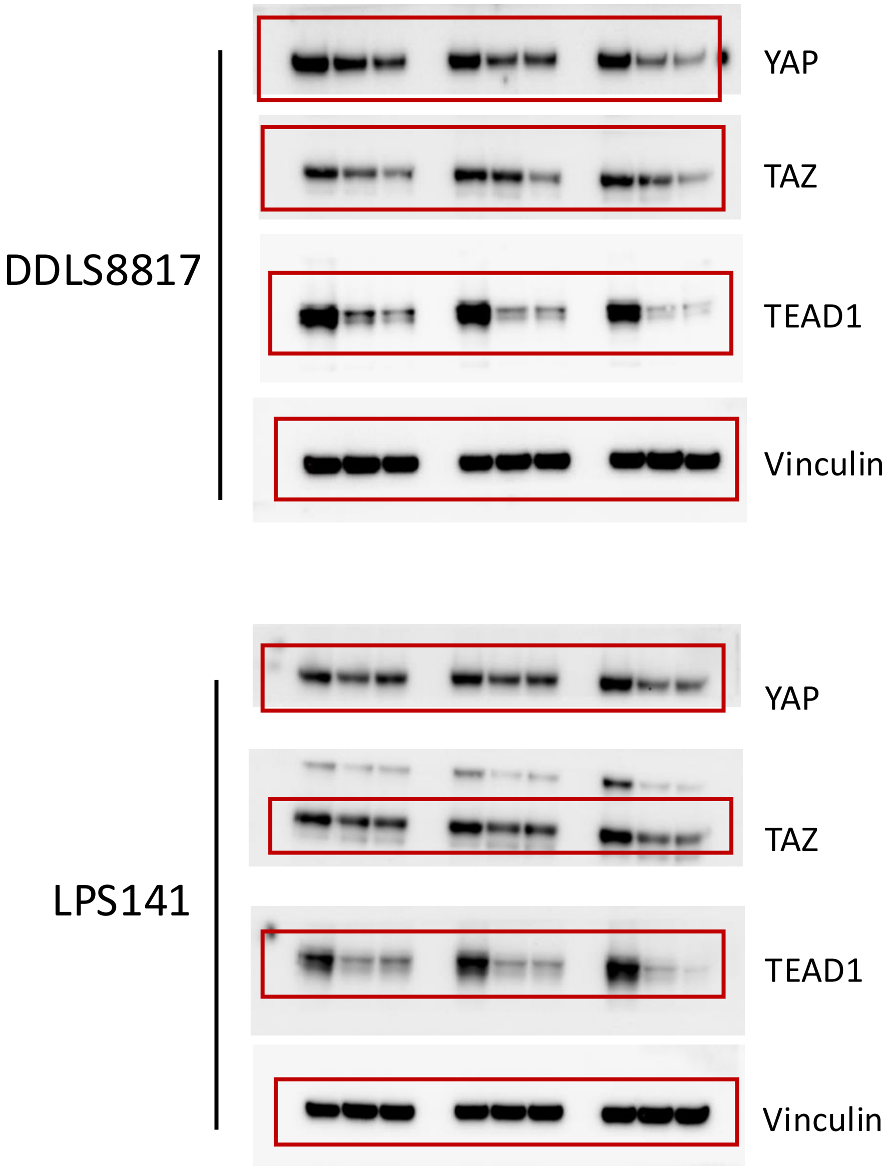
DDLS8817
YAP
TAZ
TEAD1
Vinculin
LPS141
YAP
TAZ
TEAD1
Vinculin

Figure 3B

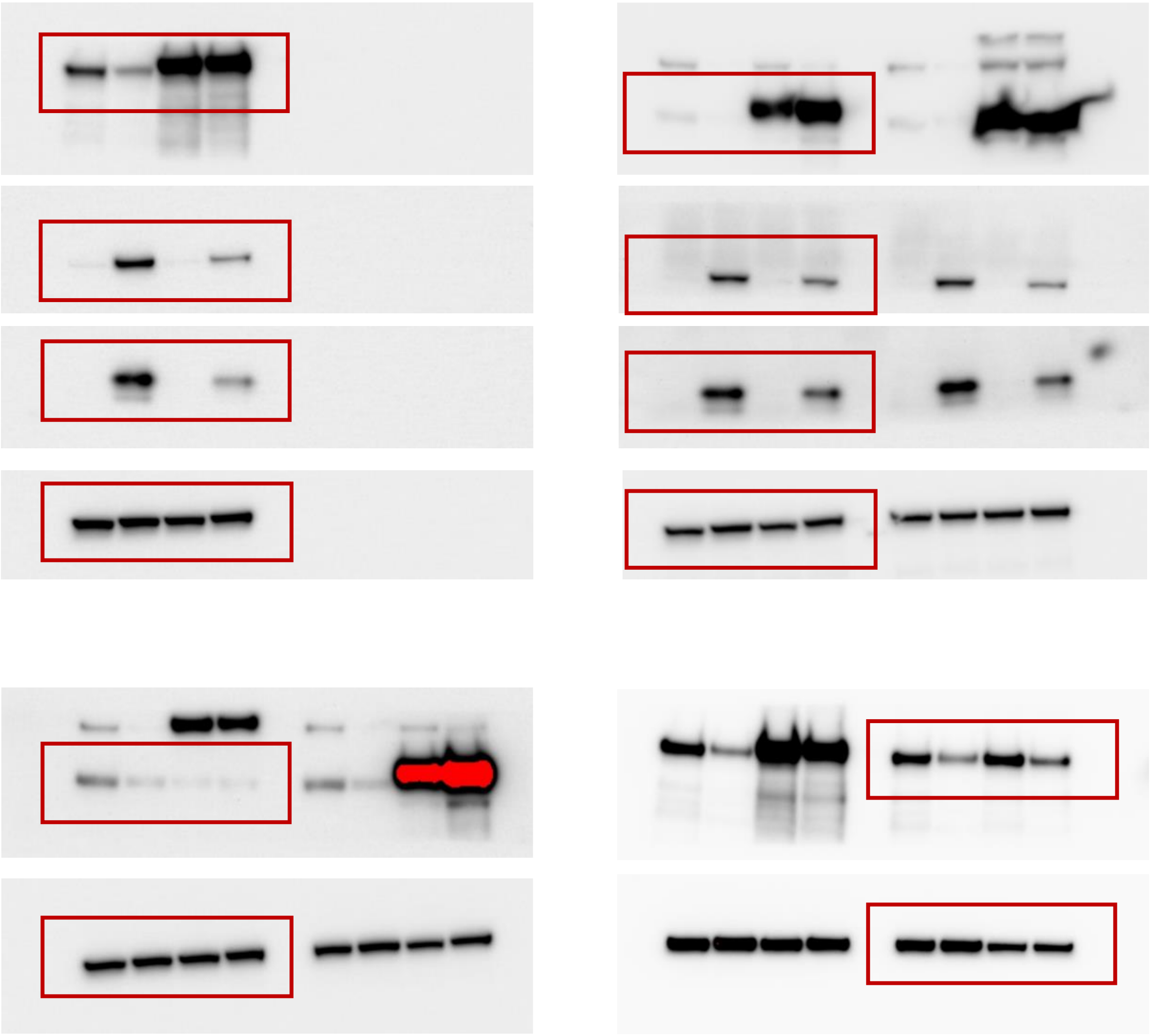

Figure 4C

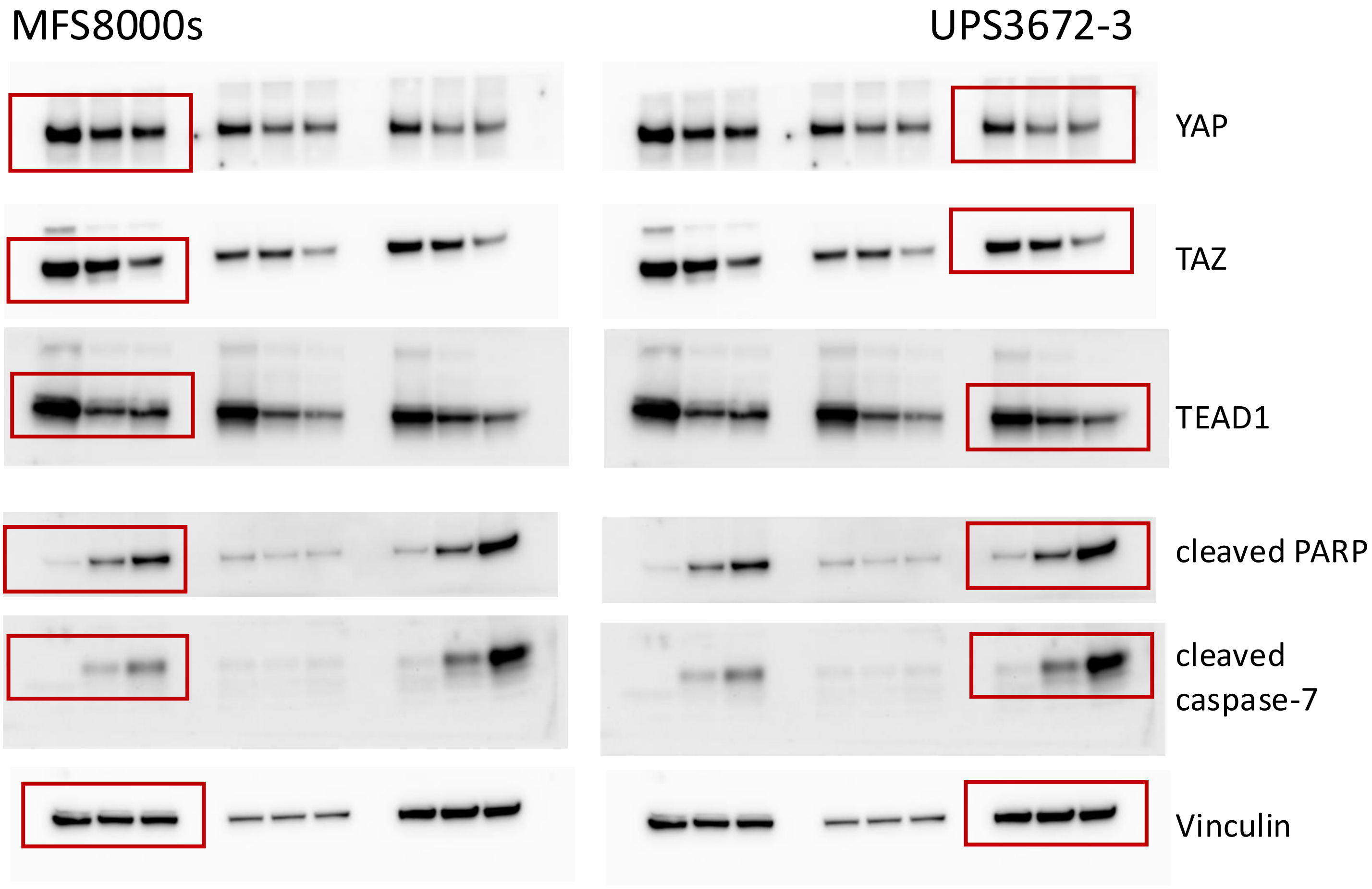
MFS8000s
UPS3672-3
YAP
TAZ
TEAD1
cleaved PARP
cleaved caspase-7
Vinculin

Figure 6A

DDLS8817

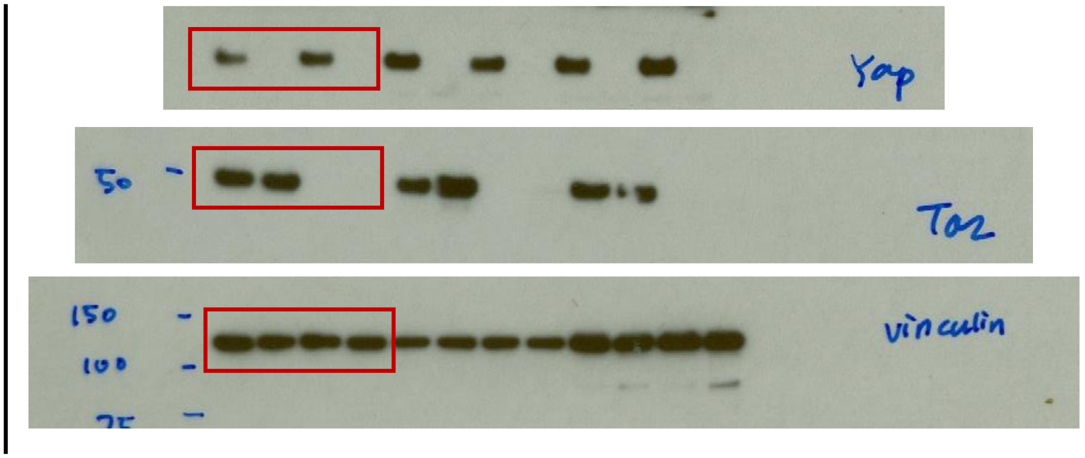

LPS141

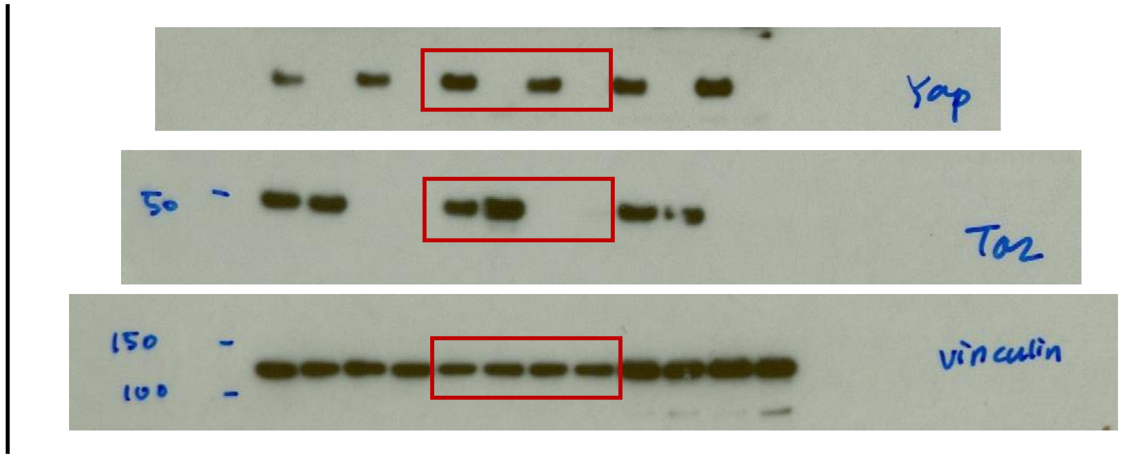

Figure 6A

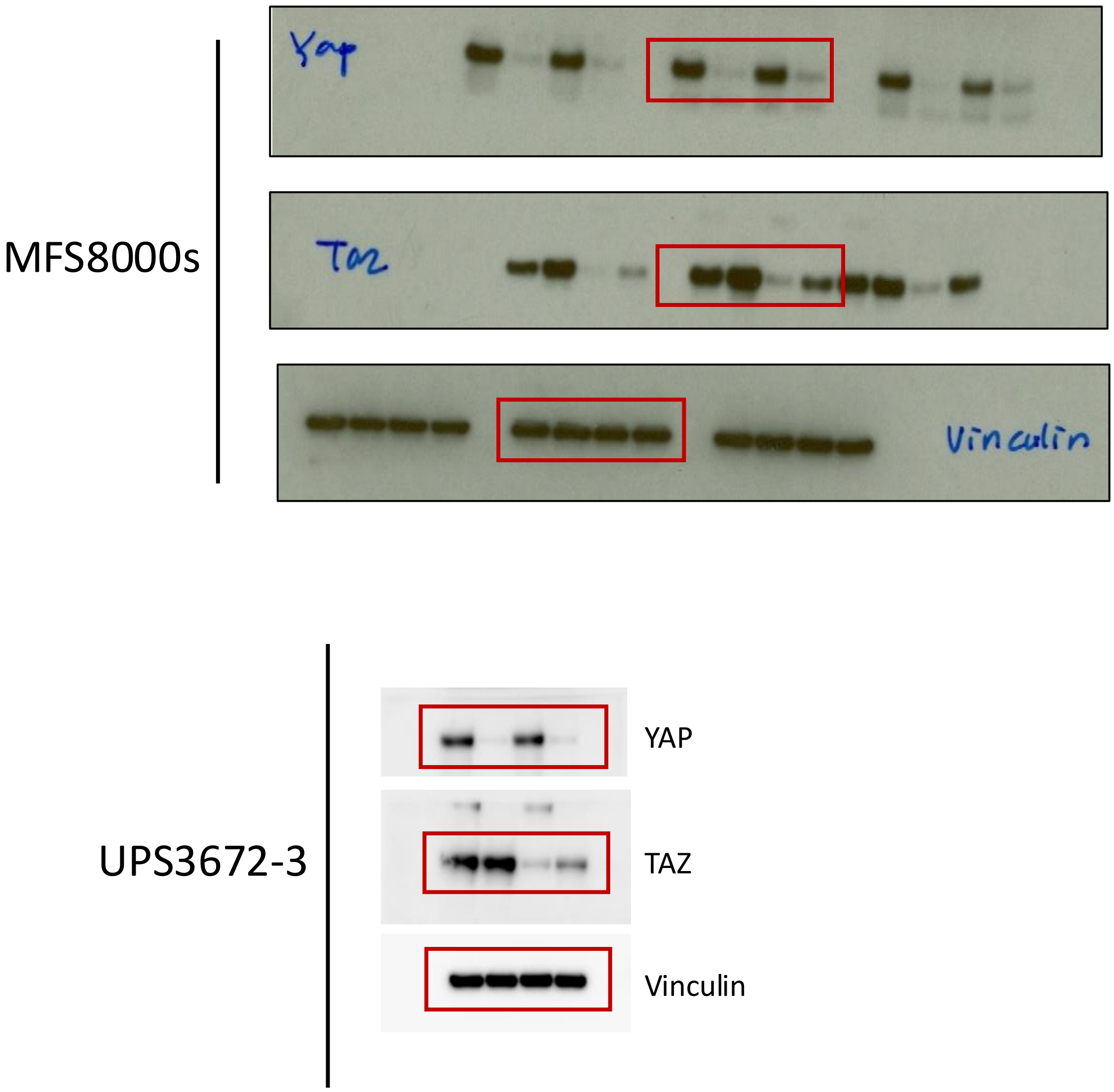

Figure 7E

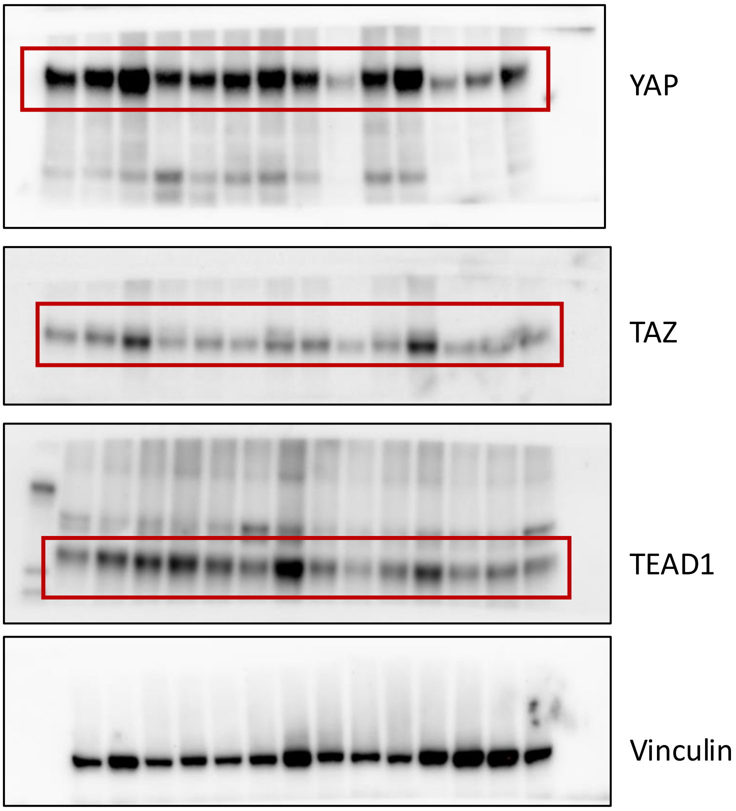

Supplementary Figure 1A

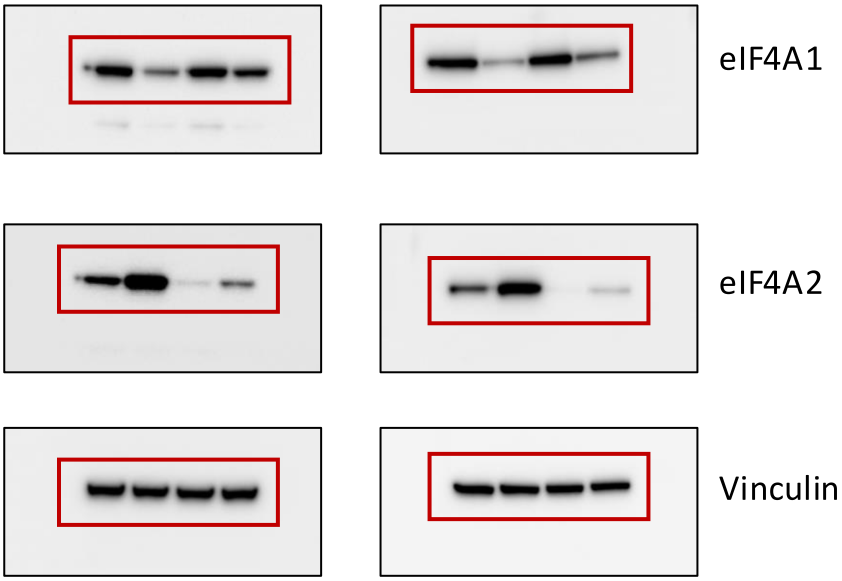

Supplementary Figure 2C

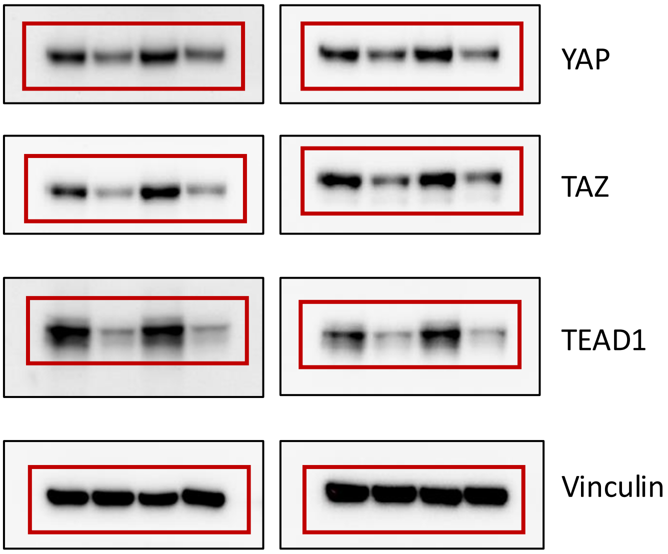

Supplementary Figure 3A

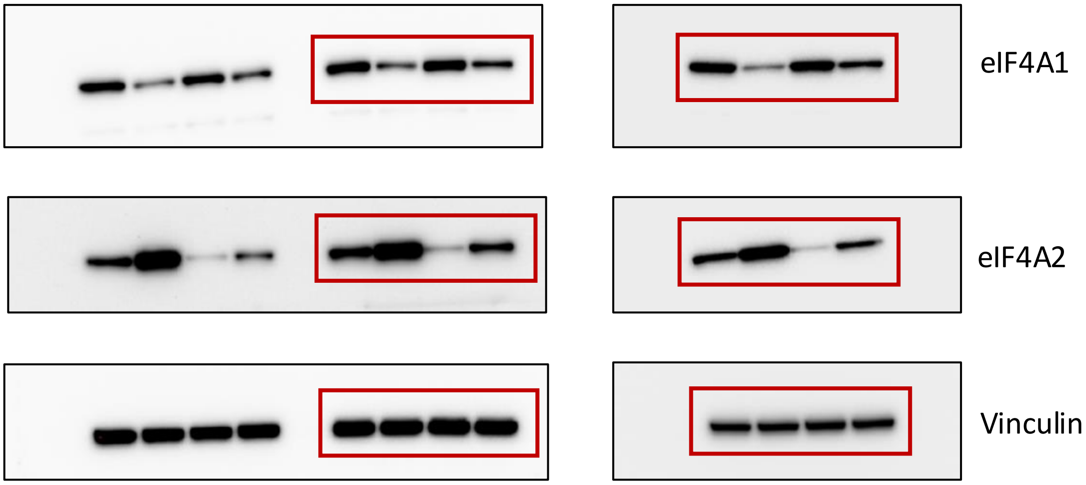

Supplementary Figure 5C

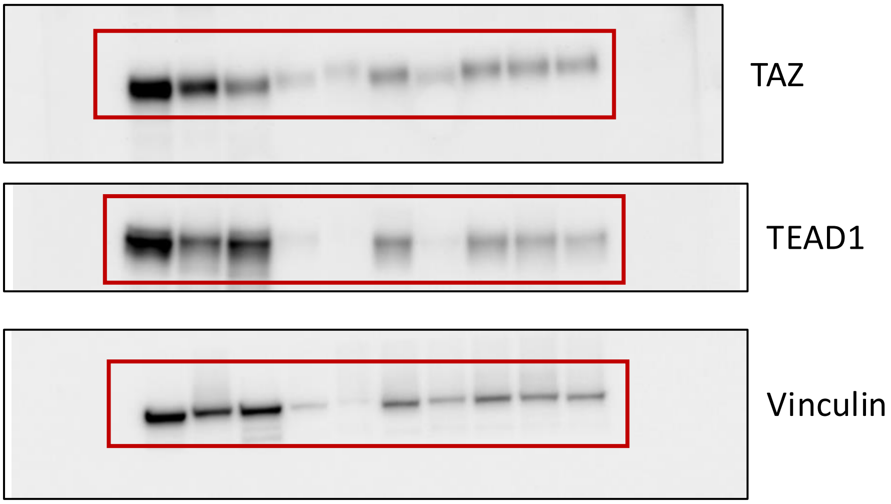

Supplementary Figure 5E

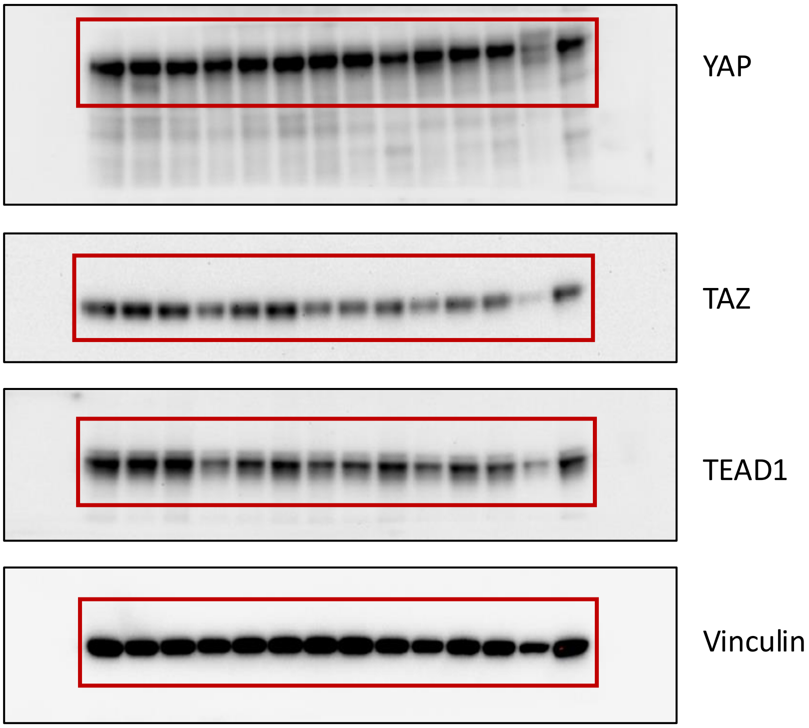

Supplementary Figure 6D

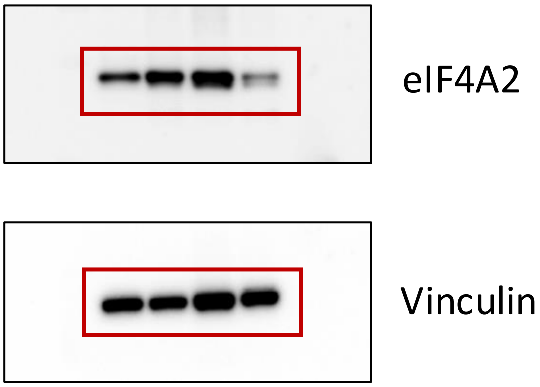

Supplement: Unedited blot and gel images [file jciinsight-11-192936-s261.pdf]
